# Experimentally altering microRNA levels in embryos alters adult phenotypes

**DOI:** 10.1038/s41598-024-63692-7

**Published:** 2024-08-16

**Authors:** Zeynep Yilmaz Sukranli, Keziban Korkmaz Bayram, Serpil Taheri, Francois Cuzin, Yusuf Ozkul, Minoo Rassoulzadegan

**Affiliations:** 1 Betul-Ziya Eren Genome and Stem Cell Center, Erciyes University, Kayseri, Turkey; 2 Department of Medical Genetics, Faculty of Medicine, Erciyes University, Kayseri, Turkey; 3 Department of Medical Genetics, Faculty of Medicine, Yıldırım Beyazıt University, Ankara, Turkey; 4 Department of Medical Biology, Faculty of Medicine, Erciyes University, Kayseri, Turkey; 5 INSERM-CNRS, Université de Nice, Nice, France

**Keywords:** miRNA, Oocyte, Blood, R-loop, Variations, Behavior, Autism, Heredity, Non-Mendelian, Retinal diseases, Diabetes complications

## Abstract

We previously identified a unique genetic feature of Autism Spectrum Disorder (ASD) in human patients and established mouse models, a low to very low level of six microRNAs, miR-19a-3p, miR-361-5p, miR-3613-3p, miR-150-5p, miR-126-3p and miR-499a-5p. We attempted to interfere experimentally in mice with two of them, miR19a-3p and miR499a-5p by microinjecting into zygote pronuclei either the complementary sequence or an excess of the microRNA. Both resulted in low levels in the tissues and sperm of the targeted microRNAs and their pri and pre precursors. This method stably modify predetermined levels of miRNAs and identify miRNA alterations that cause changes in autistic behavior and predispose the individual to an inherited disease. Excess miRNA results in single-stranded miRNA variations in both free and DNA-bound RNA (R-loop) fractions in mouse models thus appearing to affect their own transcription. Analysis of miRNAs fractions in human patients blood samples confirm low level of six microRNAs also in R-loop fractions.

## Introduction

Searching for the genetic determinant(s) of Autism Spectrum Disorder (ASD) uncovered a large number of variations in patient genomes including de novo mutations, with variable associations of the whole complex phenotype^1–4^. However, most of these genetic alterations were found in only a fraction of the patients and thus could not be primary determinants of the disease. The first and only exception has been our observation of a deviant pattern of microRNAs in the blood of 45 autistic patients in a cohort of 37 families^5^. The levels of six of them (miR-19a-3p, miR-361-5p, miR-3613-3p, miR-150-5p, miR-126-3p and miR-499a-5p), were more than 95 percent decrease in serum of affected patients’ children and reduced by about 50 % in their healthy parents and siblings. Further studies have extended the analysis to two established animal models of ASD, mice treated with valproic acid and *Cc2d1a*+*/-* heterozygotes. In both cases, the levels of miR-19a-3p, miR-361-5p, miR-150-5p, miR-126-3p and miR-499a-5p, vary as observed in the blood of human patients in the serum, hippocampus and sperm of affected animals. Altered miRNA profiles were inherited together with ASD-like symptoms in crosses with unaffected animals.

The one clearly cannot conclude on this basis of a causal relationship, although the regulatory roles of miRNAs are not excluded. Certainly, it is clear that altered expression of these miRNAs is a consistent feature in patients. To confirm the relationship between the alteration of these miRNAs and autism, we created experimental mouse models by attempting to modify the level of miRNA one by one. Microinjection of miRNA into the zygote has already been performed. MiRNA expression levels of the target gene transcripts are altered along with changes in phenotype including variations in coat color, heart size, body^6–8^ and behavior changes^9^. Above all, they have been mainly studied as regulators of mRNA translation^10^, several of them have been reported to act as regulators, interfering positively or negatively, with the transcription of target genes^11^. Experimental approaches are needed, made possible by the availability of valuable mouse models of the disease.

In this study, we attempted to stably modify predetermined profiles of miRNA with microinjection of homologous oligonucleotides into mouse zygotes. Thus, identify miRNA alterations that cause changes in autistic behavior and predispose the individual to an inherited disease. We considered miR-19a-3p as a candidate based on an in-silico search of its possible targets which indicated no less than five murine loci whose human counterparts have been associated with ASD (*Pten, Slc6a4*, *Fmr1*, *Foxp2* and *Cc2d1a*^1,12–16^. MiR-499a-5p, which was singled out by the family analysis^5^, was also retained. The construction of a mouse model of the disease allowed us to positively answer three crucial questions: (1) Which method to choose to slightly and stably modify predetermined miRNA levels, (2) to validate the link between miRNA alteration to autistic behaviors change, finally (3) does it cause a non-Mendelian hereditary disease.

## Materials and methods

### Mouse husbandry

Mice were cared for and treated according to the Principles of Laboratory Animal Care (European rules). The genetic background of mice is *Balb/c*. All animal research was performed under the relevant guidelines and regulations. All experimental protocols were approved by the Erciyes University animal ethics committee and were carried out under the license number 04-11-2012 12/54.

We confirm the study is reported in accordance with ARRIVE guidelines (https://arriveguidelines.org).

### RNA microinjection

Oligoribonucleotides synthetic miRNA were adjusted to a concentration of 1 µg/ml and microinjected into normal *Balb/c* fertilized eggs according to established transgenesis methods^8^. Oligoribonucleotides were obtained from Eurofine (sequences provided in Supplemental Table S1).

Briefly, for collecting embryos, female and male mice were mated at 3 pm. The day after, mated females were checked for the vaginal plug at 8 am and vaginal plug (+) female mice were selected. Females were sacrificed that afternoon, and their oviducts were enclosed in M2 medium (Sigma, Germany) and embryos were collected under a bino-microscope (Leica, Germany) and were transferred into M16 medium (Sigma, Germany) with a mouth pipette and incubated at 37 °C in 0.5% CO_2_ (Panasonic, Japan). To capture the pronuclei at the most prominent stage, at 2–6 pm we microinjected oligonucleotides (1–5 ng/µl, see in Supplemental Materials Table S1) with a glass pipette into the male pronucleus of embryos under an inverted microscope (Nikon, Japan). After the microinjection, the embryos were incubated at 37 °C in 0.5% CO_2_ (Panasonic, Japan). Embryos that died after microinjection under a stereo microscope (Leica, Germany) were separated and surviving embryos were transferred into M2 medium (Sigma, Germany). Living embryos were transferred to the foster mother (healthy female with a vaginal plug after mating with vasectomized male mice), under anaesthesia (10 mg/kg Xylasine as a pre-anaesthesia and 60 mg/kg Ketamine for anaesthesia) with a mouth pipette into the oviduct. Three weeks later F0 generation pups, which were born 21 days after the procedure, were separated from their mothers according to their genders and transferred to new cages and subjected to behavioral and molecular tests with other groups at two months of age.

All mice included in the study were sacrificed by cervical dislocation after behavioral experiments to avoid the influence of anesthetic substances at the molecular level. There were no other mice excluded and sacrificed in addition to this in the study.

The research conducted on autistic children, in conjunction with our team's investigations utilizing human and experimental animal models^5,17^, has revealed that autistic phenotypes manifest more frequently and severely in males compared to females. It has been demonstrated that autism spectrum traits are observed in males at a ratio of 4:1 compared to females. Furthermore, due to the possibility of hormonal irregularities in females leading to deviations in molecular-level changes, females were deliberately excluded from this study. Additionally, based on findings from our previous studies, it has been established that the autistic phenotype can be transmitted to offspring as non-Mendelian heredity^18^. Consequently, males were selected for this study.

In total, 29 F0 generation microinjected mice (5 miR19a-5p, 8 miR-19a-3p, 7 miR-499a-5p and 9 miR-499a-3p) and 53 F1 generation mice (for only behavior tests) (19 miR-19a-5p, 15 miR-19a-3p, 12 miR-499a-5p and 10 miR-499a-3p) along with 15 healthy controls were included in the study.

### Behavior tests

Behavioral experiments were started when miRNA microinjected and control group mice were 2 months of age. Each mouse underwent a single test daily between 10:00 and 16:00. Only males were tested sequentially on the same day in separate sub-sessions to allow room ventilation and cleanup. The testing tools were cleaned between trials with 70% ethanol and aerated before use. Experiments were videotaped and analyzed offline. Sociability, social-preference and object-recognition and tail suspension tests were analyzed using “EthoVision 9” software (Noldus, Wageningen, Netherlands). Marble-burying tests were analyzed manually by an observer blind to the group of the mice.

#### Novel object recognition test

A mouse-size object and a second identical object were placed in a square box with an open top with lines dividing it at the base and a wall enclosing it. A mouse-sized object and a second identical object are placed. The mouse’s proximity to the objects and the number of visits were counted on the first day. On the second day, one of the objects was replaced with a new object. The discriminating index, the number of times the mouse approached both unfamiliar and recognized objects, and their proximity were all measured. The data obtained is defined as the discrimination index when the difference between the total time spent with the new object and the total time spent with the familiar object is divided by the total duration and multiplied by 100. The proportion of the mice’s overall interaction time with the new object is measured. The learning and memory abilities of mice are examined in this experiment. Because mice have an innate desire for novelty, it was anticipated that they would spend more time with the novel object to discover more about it^16^.

#### Social Interaction test

The Social Test assesses cognition in mouse models of Central Nervous System (CNS) disorders by evaluating general sociability and interest in social novelty. Since they are sociable creatures, rodents normally prefer to spend more time with other rodents (sociality) and are more likely to approach a stranger than a friend (social novelty). The rectangular, three-compartment box used in Crawley's friendliness and social novelty test consists of a rectangular three-compartment box. Every room is 19 × 45 cm and is a system with partitioned walls and a central chamber made of clear plexiglass that allows open access to every area^19^. A mouse is initially placed into a middle compartment for 5 min while the other compartment was left empty. Under the parameters found to be connected to the mouse in the chamber, data were generated using the EthoVision system, and statistical analysis was performed along with comparisons to the control group.

#### Marble burying test

The marble burying test is commonly used to evaluate rodent neophobia, which includes shyness around unfamiliar objects, anxiety and obsessive-compulsive or repetitive behaviors^20^. We positioned the bedding that we usually use to care for our mice empty cage at the height of 5 cm in an empty cage. There are five rows of four marbles each, totaling twenty marbles. The experimental mouse was let out of one corner of the cage and given 30 min to roam around. The mouse was removed from the cage after 30 min, and the number of balls discovered under the bedding was tallied and recorded. The number of embedded balls was used for statistical analysis.

#### Tail suspension test

The mouse suspended by the tail test is a model experiment model of autism on observing that after initial escape-directed movements, mice develop a sedentary stance when placed in an unavoidably stressful situation. The stressful situation during tail-hanging includes the hemodynamic stress of hanging, and autistic models seem to have reduced mobility and escape abilities^21^. The experimental setup was designed to simultaneously test three different mice. Thick cardboard-like sheets measuring 25 cm high were cut and placed between the mice so that they could not see each other. Since the mice are white in color, a black background was used. Twelve cm-long tapes were cut and hung in the experimental setup by sticking them to the tail ends of mice in such a way that their tails would not be damaged. By watching recordings with a video camera, the mobility and immobility time of mice were calculated for 6 min. The immobility time was used for statistical analysis.

After the behavioral experiments of the F0 generation were completed, two females and one male were mated to obtain the F1 generation. When obtaining the F1 generation, the F0 generation and the control group were sacrificed and blood, hippocampus and sperm samples were taken. For the F1 generation, behavioral experiments were conducted when they were 2 months old. The F1 generation was only studied for behavioral changes and molecular analysis were not performed.

### RNA Isolation from tissue

Hippocampus and blood samples were taken into 500 µl Purezol (Biorad, USA, CA, Cat No: 7326890) and the hippocampus was homogenized with a two ml syringe. Subsequently, total RNA isolation was carried out according to the manufacturer's instructions. 200 µl of chloroform was added to the homogenized tissues and blood and centrifuged at + 4 °C at 12.000 g for 20 min. The aqueous phase was taken and isopropanol was added in a ratio of 1:1. It was centrifuged + 4 °C for 10 min at 12.000 g. One ml of 75% ETOH was added to the supernatant and then centrifuged at 7500 g at + 4 °C. This process was repeated twice; and the pellets were incubated at room temperature to dry. The pellets were resuspended in 30 µl of nuclease-free water (Qiagen, Germany, Center Mainz, Cat No: 129114). Prior to working with them total RNAs were stored at − 80 °C.

For sperm RNA isolation, sperm were extracted from vas deferens canals of mice, which were thoroughly turned upside down and shaken for 5 min and then centrifuged at 1000 rpm for 2 min. The supernatant was taken into a new falcon and centrifuged at 3000 rpm for 15 min. The pellets were resuspended in 500 µl of dH_2_O was and after 5 min, PBS was added to make up to 5 ml. After centrifugation at 3000 rpm for 15 min, the supernatant was discarded. 3 µl DTT (1 M) and 1000 µl Purezol (Biorad, USA, CA, Cat No: 7326890) were added to the pellets in each falcon tube which were kept on ice for 5 min. The resuspended pellets were transferred to 1.5 Eppendorf tubes, homogenized with an injector, and 200 µl of chloroform was added. After centrifuging at 12.000 g for 15 min at + 4 °C, an equal volume of isopropanol was added to the aqueous phase which was then incubated at − 20 °C for 30 min and centrifuged at 12.000 rpm for 30 min. The supernatants were discarded and 1 ml of 70% ETOH was added to each pellet which were centrifuged at 7500 rpm for 10 min. After discarding the supernatants, 500 µl dH_2_O, 25 µl 5M NaCl and 1 ml 100% ETOH were added to each sample, which were incubated at − 20 °C overnight and subsequently centrifuged at 10,000 rpm for 30 min. 1 ml of 70% ETOH was added to each pellet followed by centrifuged at 10,000 rpm for 10 min. The final pellets were resuspended in 50 µl of nuclease-free water.

### Blood collection and serum separation

Blood samples were collected from the founder mice between 11.00 am and 13.00 to eliminate unwanted parameters. All protocols for serum separation were completed within 1 h of drawing blood. Serum was separated with centrifugation at 3500 rpm for 10 min at room temperature. Hemolyzed samples were excluded from the study. The clear supernatant was collected into new RNase/DNase-free microfuge tubes in 200 μl aliquots. RNA was isolated using a High Pure miRNA Isolation Kit (Roche, Mannheim, Germany) according to the manufacturer’s instructions and stored at − 80 °C until use.

The same experimental protocol was used for blood collection and serum separation from patients and human control, all parents gave written informed consent before participation (09-20-2011 committee number: 2011/10).

### DNA-bound R-loop hybrids isolation

DNA-bound RNA hybrids in R-loop were isolated with the Quick-DNA/RNA™ Microprep Plus Kit from blood, hippocampus and sperm samples. Manufacturer’s instructions were followed for isolation and DNAse I treatment was applied to release RNA from DNA/RNA hybrids. See publication Rassoulzadegan et al.^22^ for all controls: 1-RNase H treatment (RNase specific for the degradation of attached RNA in DNA hybrids) from which all signals are eliminated, 2-RNaseA treatment which only degrades free RNA and RNA hybrids /DNA remain intact.

### cDNA preparation

Isolated RNA samples were reverse-transcribed into cDNA in 20 μl final reaction volumes using miScript II RT Kit (Qiagen, Germany) as specified in the manufacturer’s protocol. Reverse transcription was performed using the SensoQuest GmbH Thermal Cycler (Göttingen, Germany). cDNA samples were kept at − 80 °C until PCR analysis.

### Quantitative real-time polymerase chain reaction (qRT-PCR)

QRT-PCR was performed by using miScript SYBR® Green PCR Kit (Qiagen, Hilden, Germany Cat No: 218073) with the high-throughput Light Cycler 480 II Real-Time PCR system (Roche, Germany, Mannheim). cDNA samples were diluted with Nuclease Free Water (1:5). The reaction was performed according to manufacturer’s instructions. About 10 μl Syber Green Master Mix, 2 µl 10x Universal Primer, 2 µl primer assays (see in Table S1 and S2) and 4 µl nuclease free water mixed and pipetted into a 96 well plate as 18 µl and 2 μl of 1:5 diluted cDNA was pipetted into each well and mixed. The Real-Time PCR step was performed by using the Light Cycler 480 II Real-Time PCR system with the following protocol: thermal mix followed by the Activation step at 95 °C for 15 min, then a denaturation step at 94 °C for 15 s followed by an annealing step at 55 °C for 30 s followed by an extension step at 70 °C for 30 s. After activation step, all steps were carried out for 40 cycles.

### Statistical analysis

After the results were obtained, experimental groups and control groups comparisons were made. The compliance of the data to normal distribution was evaluated by the histogram, q–q graphs and Shapiro–Wilk test. Statistical analysis was performed by two-tailed, one-way analysis of variance (ANOVA), uncorrected Fisher’s least significant difference (LSD). Kruskal Walls, student t-test and Mann Whitney-U test depending on whether the data showed normal distribution or not. Data were analyzed using SPSS version 22 (IBM, USA) and Graph-Pad Prism 8.0 software. Results with *p* values < 0.05 were considered statistically significant. Data are expressed as the mean with SD.

### Ethical approval

All experimental protocols were approved by the Erciyes University Animal Ethics Committee and were carried out under the license number 04-11-2012 12/54.

## Results

### Changing the amount of a given miRNA from the zygote to the mouse: miRNAs microinjection into zygote results in complex but reproducible heritable changes in adult tissues

**Figure 1:**
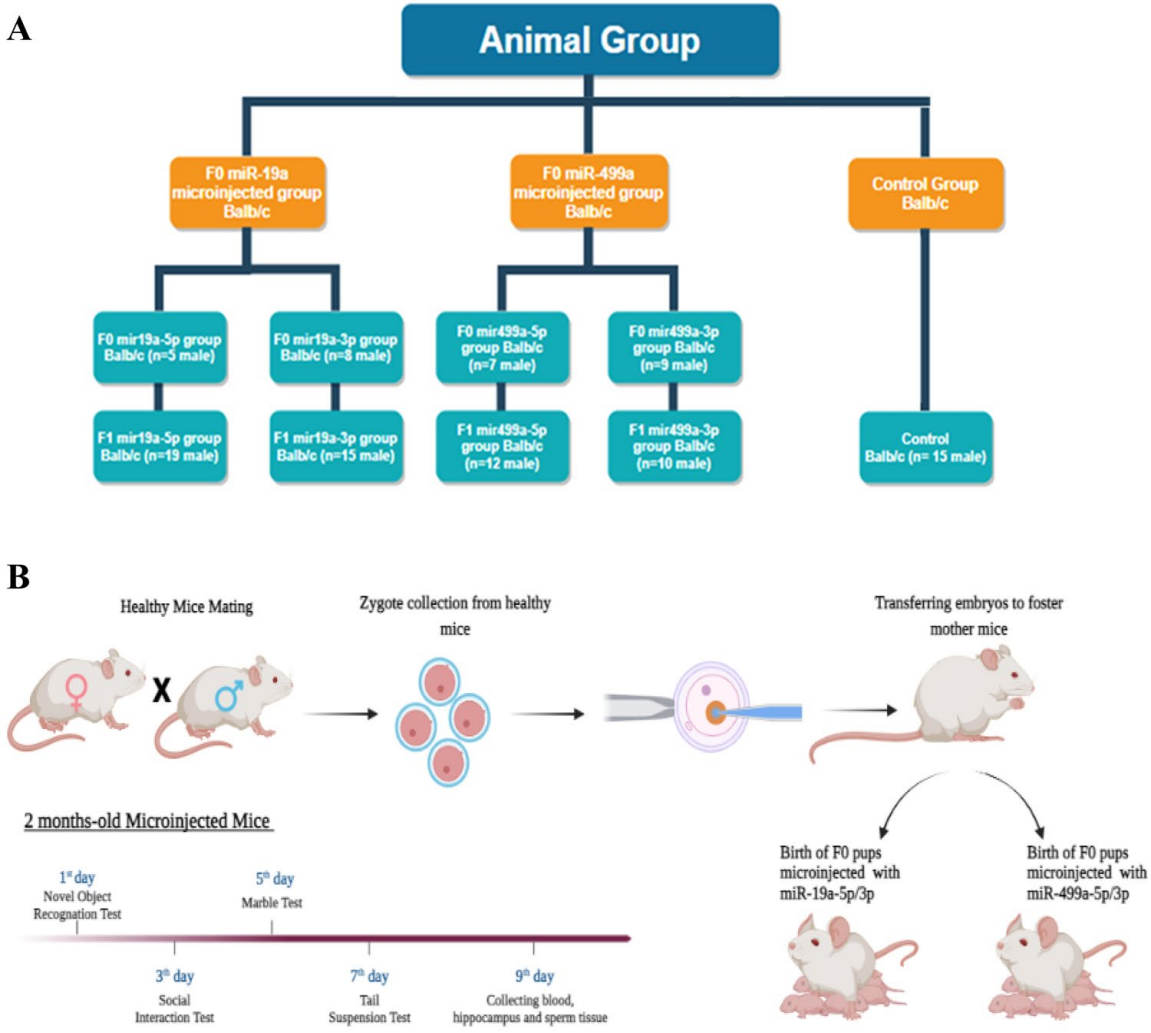
Experimental timeline (**A**) Mice group (**B**) Steps from collection, manipulation and embryos transfer to mouse birth and behavioral tests.

**Figure 2:**
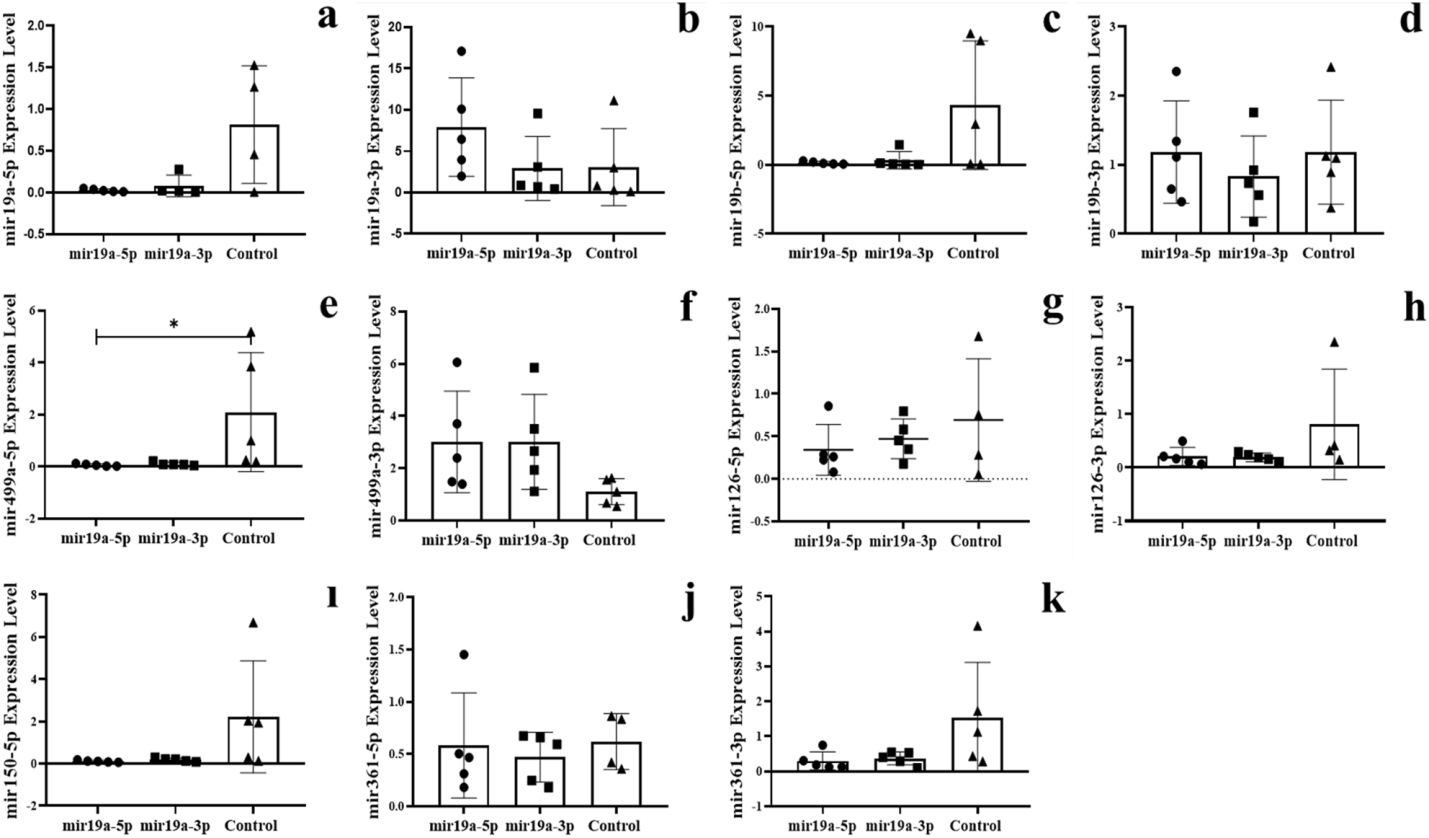
The expression of miRNAs in the blood of F0 genaration mice born after microinjection of miR-19a (5p/3p) into fertilized mouse eggs. (**a)** miR-19a-5p, (**b**) miR-19a-3p, (**c**) miR-19b-5p, (**d**) miR-19b-3p, (**e**) miR-499a-5p, (**f**) miR-499a-3p, (**g**) miR-126a-5p, (**h**) miR-126a-3p, (**ı**) miR-150a-5p, (**j)** miR-361a-5p and (**k**) miR-361a-3p (All data were expressed with mean and ± SD; (N) = 5 for F0 mir19a-5p group, (N) = 5 for F0 miR19a-3p group and (N) = 5 for control; The x-axis illustrates the groups formed by microinjection with miR-19a-5p or -3p, alongside the control group. The y-axis represents the expressions of the miRNAs utilized in the study).

**Figure 3:**
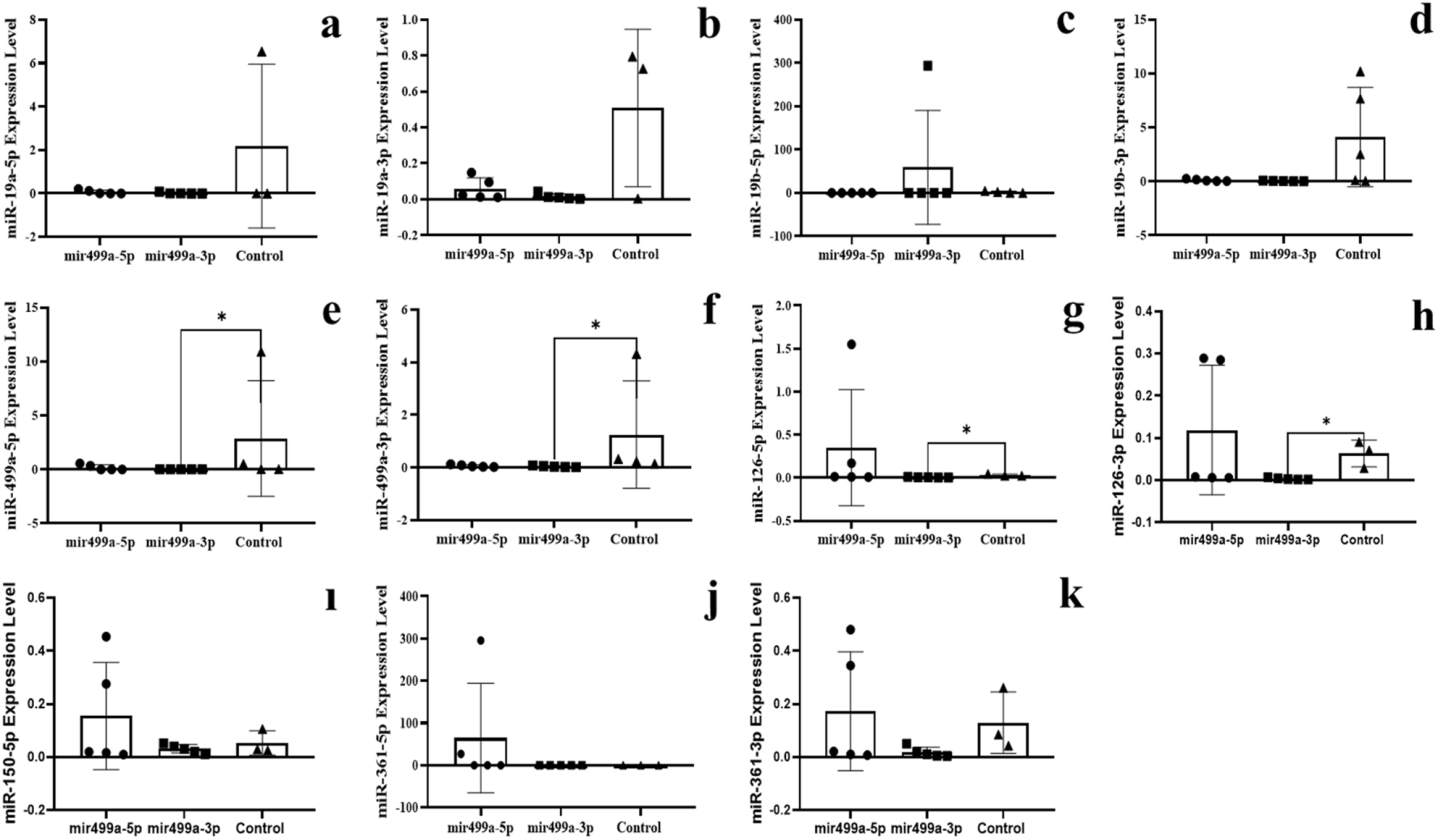
The expression of miRNAs in the blood of F0 generation mice born after microinjection of miR-499a (5p/3p) into fertilized mouse eggs. (**a**) miR-19a-5p, (**b**) miR-19a-3p, (**c**) miR-19b-5p, (**d**) miR-19b-3p, (**e**) miR-499a-5p, (**f**) miR-499a-3p, (**g**) miR-126a-5p, (**h**) miR-126a-3p, (**ı**) miR-150a-5p, (**j**) miR-361a-5p and (**k**) miR-361a-3p (All data were expressed with mean and ± SD; (N) = 5 for F0 mir499a-5p group, (N) = 5 for F0 miR499a-3p group and (N) = 5 for control; The x-axis illustrates the groups formed by microinjection with miR-499a-5p or -3p, alongside the control group. The y-axis represents the expressions of the miRNAs utilized in the study).

To test whether microinjection of a microRNA into a zygote would result in altered expression of the same miRNAs in adult, we first examined mice derived from zygotes microinjected with the complementary sequence of the two microRNAs studied (Fig. 1 and Supplementary Table 1). We microinjected an excess of the sequence complementary to the -3p miRNA strand (see Figs. 2 and 3). Since the models created showed a decrease in the level of miRNA in the blood with the -3p strand microinjected into the egg, we again created complementary models, with microinjection of the -5p strand of the same miRNA. Unexpectedly results show equivalent downregulation of -5p and -3p miRNAs in the blood of these mice.

A summary of mice born after microinjection and miRNAs analyses is listed in Fig. 1.

The expression levels of six miRNAs (-3p and -5p strand) were analyzed in two fractions of RNA preparation (see Method section). The first results presented concern the free fraction with standard extraction protocol of RNA after microinjection of a specific miRNA in Figs. 2 and 3. The results showed the expected decrease after microinjection of the complementary sequence -3p but also by microinjection of an excess of the strand of miRNA -5p itself.

All together, our results indicate that microinjection of miRNAs (-3p and/or -5p strand) modifies their own expression in different tissue and could influence not only its reverse sequences but also other microRNA levels (listed here, see Figs. 2 and 3). For example: miR-19a and miR-19b are transcribed from a polycistronic locus^13^. Whether variations on strand miR-19a are seen as well on miR-19b? Microinjection of miR-19a (-5p and/or -3p) was found to downregulate the levels of miR-19a-5p (Fig. 2a). While miR-19a-3p (Fig. 2b) are unchanged by miR-19a-3p and or upregulated by the miR-19a-5p strand. MiR-19b is transcribed into polycistron with miR-19a located on the same chromosomal locus thus follow the same variations as miR-19a, see for miR-19b-5p (Fig. 2c), and miR-19b-3p (Fig. 2d) transcripts. On the other hand, five others miRNAs are located on different chromosomes. Of the other miRNAs- (see Table S1) miR-499-5p (Fig. 2e), miR-150-5p (Fig. 2i), miR-126-3p (Fig. 2g) and miR-361-3p (Fig. 2h) are downregulated but miR-499-3p (Fig. 2f) is up-regulated.

In mice microinjected with miR-499a-3p, four microRNAs were downregulated compared to controls: miR-499a-5p (Fig. 3e), miR-499a-3p (Fig. 3f), miR-126-5p (Fig. 3g) miR-126-3p (Fig. 3h). The microinjection results validate the multiple effects of miR19a-3p on miR499-3p (up-regulation) and miR-499a-5p (downregulation) and the subsequent effect of miR-499a-5p and miR-499a-3p on its own transcription and also mainly on miR-126-3p and miR-126-5p (Fig. 3g and h).

These results support our first assumption that exposure of zygotes to extra-miRNAs permanently affects miRNAs levels (here, five mi*RNA*s) in mouse blood cells.

### Downregulation of the microRNAs at the transcription level

**Figure 4:**
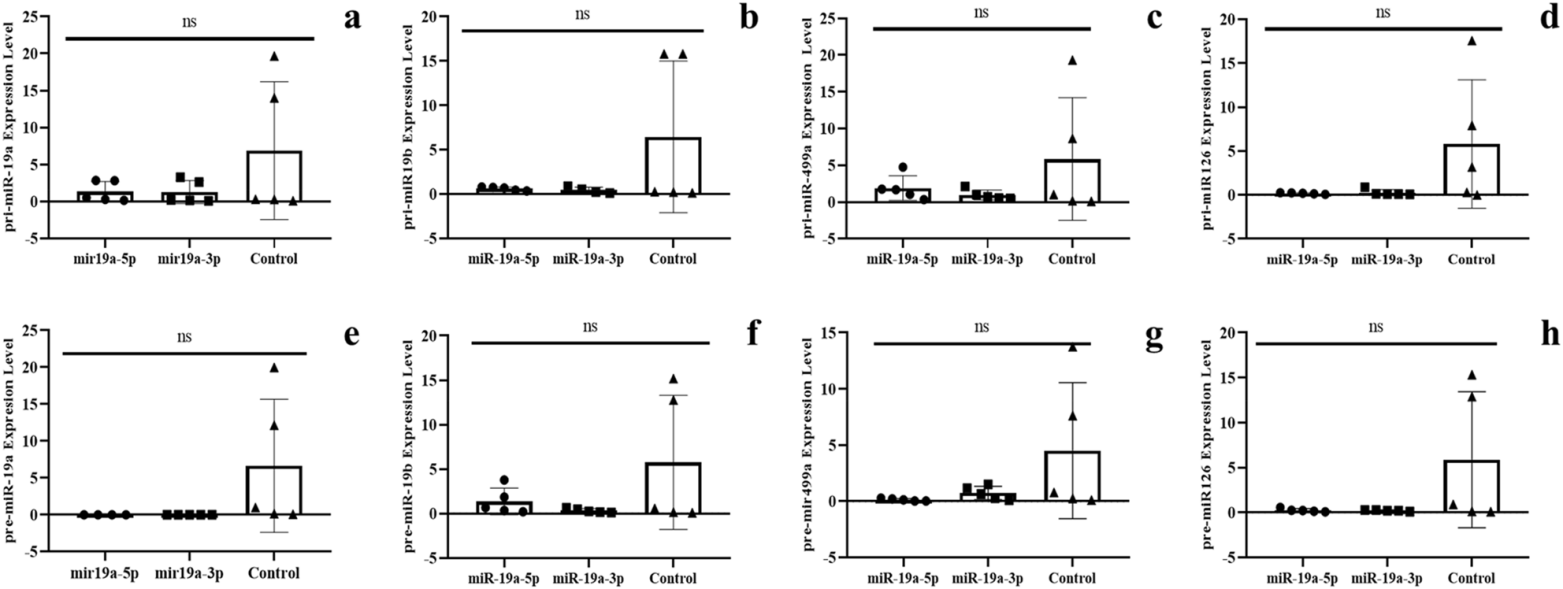
The expression of pri and pre-miRNAs in the blood of F0 generation mice born after microinjection of miR-19a into fertilized eggs. (**a**)* pri-miR-19a, *(**b**)* pri-miR-19b, *(**c**)* pri-miR-499a, *(**d**)* pri-miR-126a, *(**e**)* pre-miR-19a, *(**f**)* pre-miR-19b, *(**g**)* pre-miR-499a and *(**h**)* pre-miR-126a* (All data were expressed with mean and ± SD; (N) = 5 for F0 mir19a-5p group, (N) = 5 for F0 miR19a-3p group and (N) = 5 for control; The x-axis illustrates the groups formed by microinjection with miR-19a-5p or -3p, alongside the control group. The y-axis represents the expressions of the miRNAs utilized in the study).

**Figure 5:**
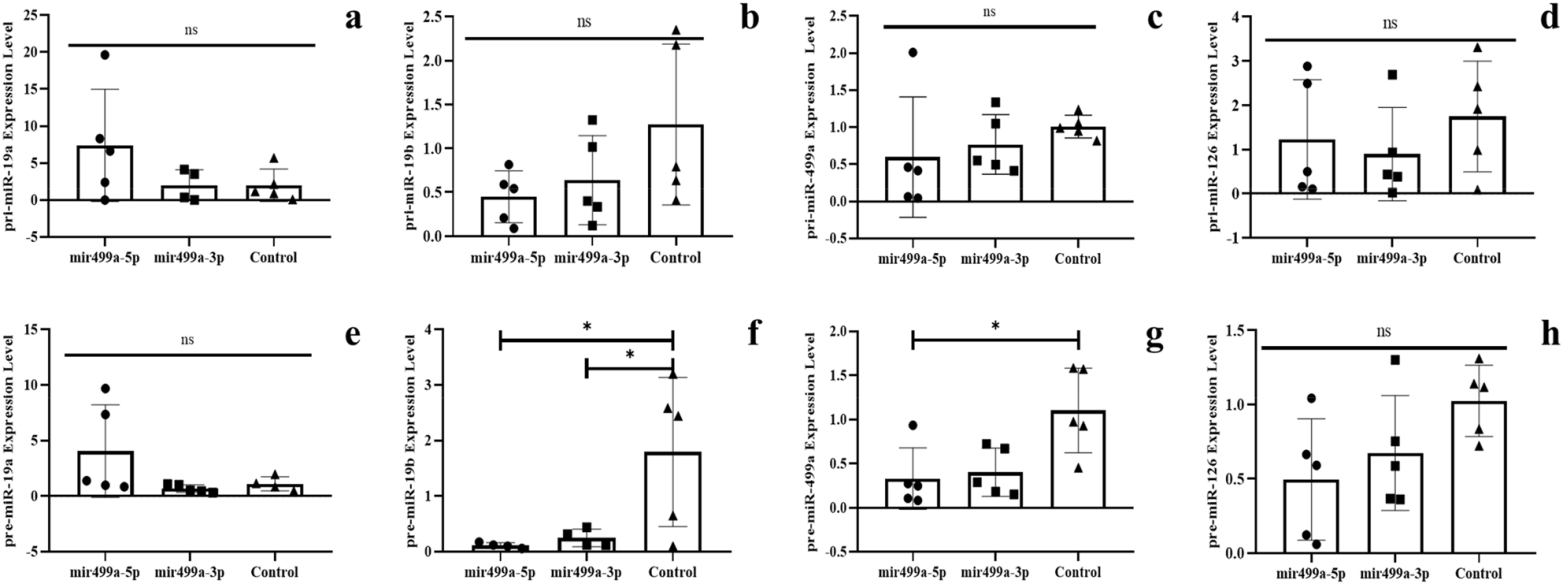
The expression of pri and pre-miRNAs’ in the blood of F0 generation miceborn after microinjection of miR-499a into fertilized mouse eggs. (**a**)* pri-miR-19a, *(**b**)* pri-miR-19b, *(**c**)* pri-miR-499a, *(**d**)* pri-miR-126a, *(**e**)* pre-miR-19a, *(**f**)* pre-miR-19b, *(**g**)* pre-miR-499a and *(**h**)* pre-miR-126a* (All data were expressed with mean and ± SD; (N) = 5 for F0 mir499a-5p group, (N) = 5 for F0 miR499a-3p group and (N) = 5 for control; The x-axis illustrates the groups formed by microinjection with miR-499a-5p or -3p, alongside the control group. The y-axis represents the expressions of the miRNAs utilized in the study).

The microRNAs are transcribed by RNA polymerase II (RNAP II), and the products (Pri-miRNAs) are converted into Pre-miRNAs by Drosha-DGCR8. Next, we examined the levels of immature miRNA transcripts. At this point, our analyses used separate specific oligonucleotides (see Table S2 for oligonucleotide sequences) and RT q-PCR to amplify transcript fragments specific for Pri- or Pre- miRNAs accumulation. As shown in Figs. 4 and 5 from results in blood and Supplementary Figures in the hippocampus (see in Figure S1 for mature miRNA expression and Figure S2 for expression of Pri- and Pre- miRNA), miRNAs transcripts Pri- and Pre- are altered in all tissues after microinjection of mature mi*RNA*s into the zygote.

In fact, in blood samples from mice, microinjected with mir-19a-5p or -3p downregulation of miR-19a pri (Fig. 4a) and pre transcripts is also observed (Fig. 4e). Similarly, microinjected miR-19b samples exhibit downregulation of miR-19b pri (Fig. 4b) and pre (Fig. 4f) transcripts. In the case of miR-499a, pri and pre transcripts are downregulated (Figure c and g respectively), while mature miR-499a-3p is up-regulated. Pri and pre-miR-126a transcripts are consistently downregulated, as well as in mature miR-126a-5p and miR-126a-3p (Fig. 4d and h respectively).

On the other hand, Fig. 5 although microinjected with miR-499a-5p pri and pre-miR-19a are up-regulated (Fig. 5a and e), while their levels are unchanged with miR-499a-3p (Fig. 5a and e). MiR-19b microinjected samples show downregulation in miR-19b pri and pre transcripts (Fig. 5b and f). For miR-499a group, pri-miR499a has no significant distinction (Fig. 5c), but pre-miR499a is downregulated (Fig. 5g). In pri-miR126a transcripts, no discernible differences are noted (Fig. 5d), while pre-miR-126a is downregulated (Fig. 5h). In Figure S2 (hippocampus) and S3 (sperm) are shown results of pri and pre-miRNAs levels. These findings provide insights into the dynamics of networks within molecular regulation.

A summary of results are shown in Figure S4.

Modification of the levels of the Pri- and Pre- transcripts supports the notion of a transcriptional regulatory mechanism. Since, five out of the six microRNAs are located on different chromosomes and positions the question of common transcription regulation is not examined here, which will require further investigation.

### Does a decrease in miRNA distribution play a causal role in behavior? Behavior analysis reveals modified animals

We have previously reported that in human patients and animal models, downregulation of miR-19a-3p, miR-361-5p, miR-150-5p, miR-126-3p and miR-499a-5p is part of behavioral component switching. At that time, it was not possible to establish whether the microRNAs decrease could be a sufficient causal element in the initiation of behavioral variations. Since we are now able to alter the profile of miRNAs in otherwise healthy organisms, it becomes possible to determine whether altering miRNAs triggers at least part of the behavioral changes.

Behavior tasks were performed on male mice from two months of age by recognition of novel objects as well as familial and social interactions assays performed by tail suspension and marble burying tests (see timeline in Fig. 1).

**Figure 6:**
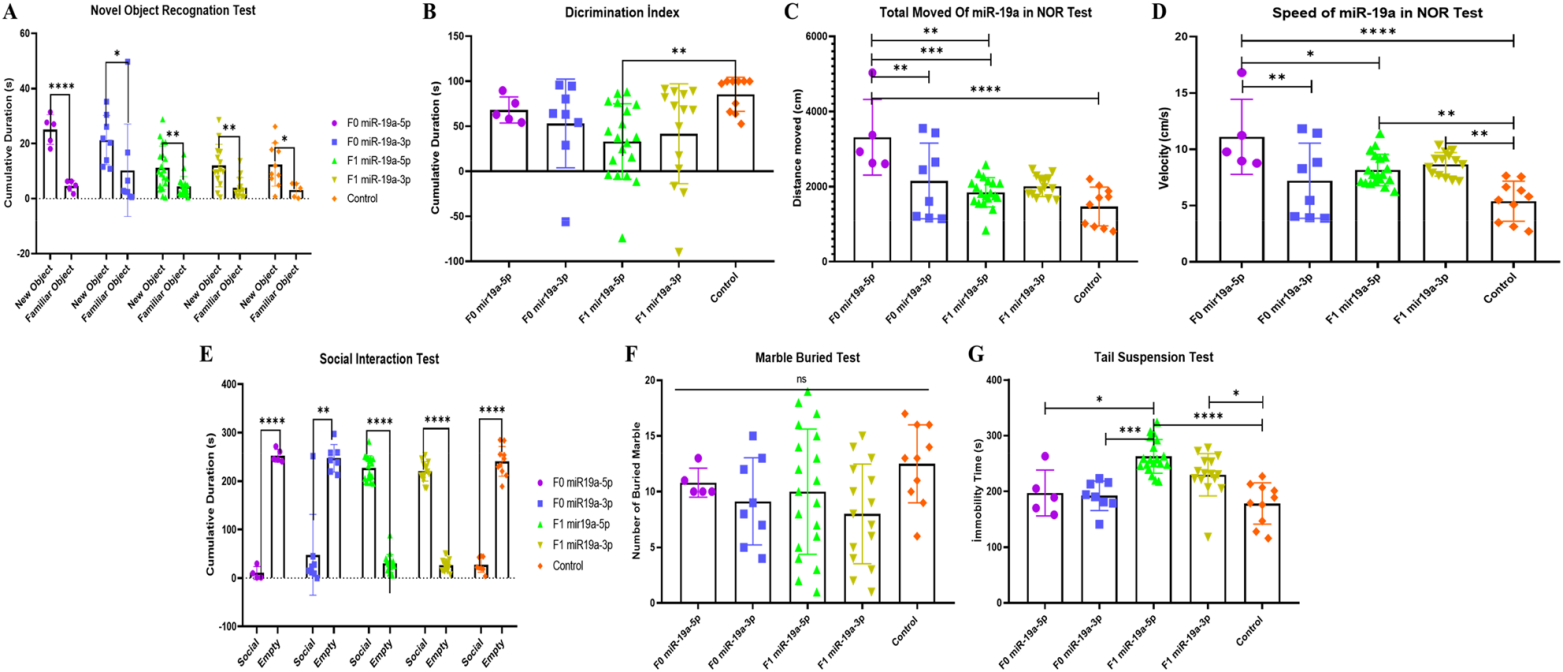
Behavior tests result of F0 generation mice group born after microinjection mir19a (5p/3p) into fertilized mouse eggs (**A**) Comparison of total exploration time between new object and familiar object, (**B**) Comparison of total exploration time in F0 and F1 generations in NOR (**C**) Comparison of total movement between F0 and F1 generations in NOR test, (**D**) Comparison of total speed in NOR test, (**E**) Comparison of total social time, (**F**) Comparison of total buried number in F0 and F1 generations in Marble Test and (**G**) Comparison of immobility time in F0 and F1 generations in Tail Suspension Test. (All data were expressed with mean and ± SD; (N) = 5 for F0 mir19a-5p group, (N) = 8 for F0 miR19a-3p group, (N) = 19 for F1 mir19a-5p group, (N) = 15 for F1 miR19a-3p group and (N) = 15 for control).

**Figure 7:**
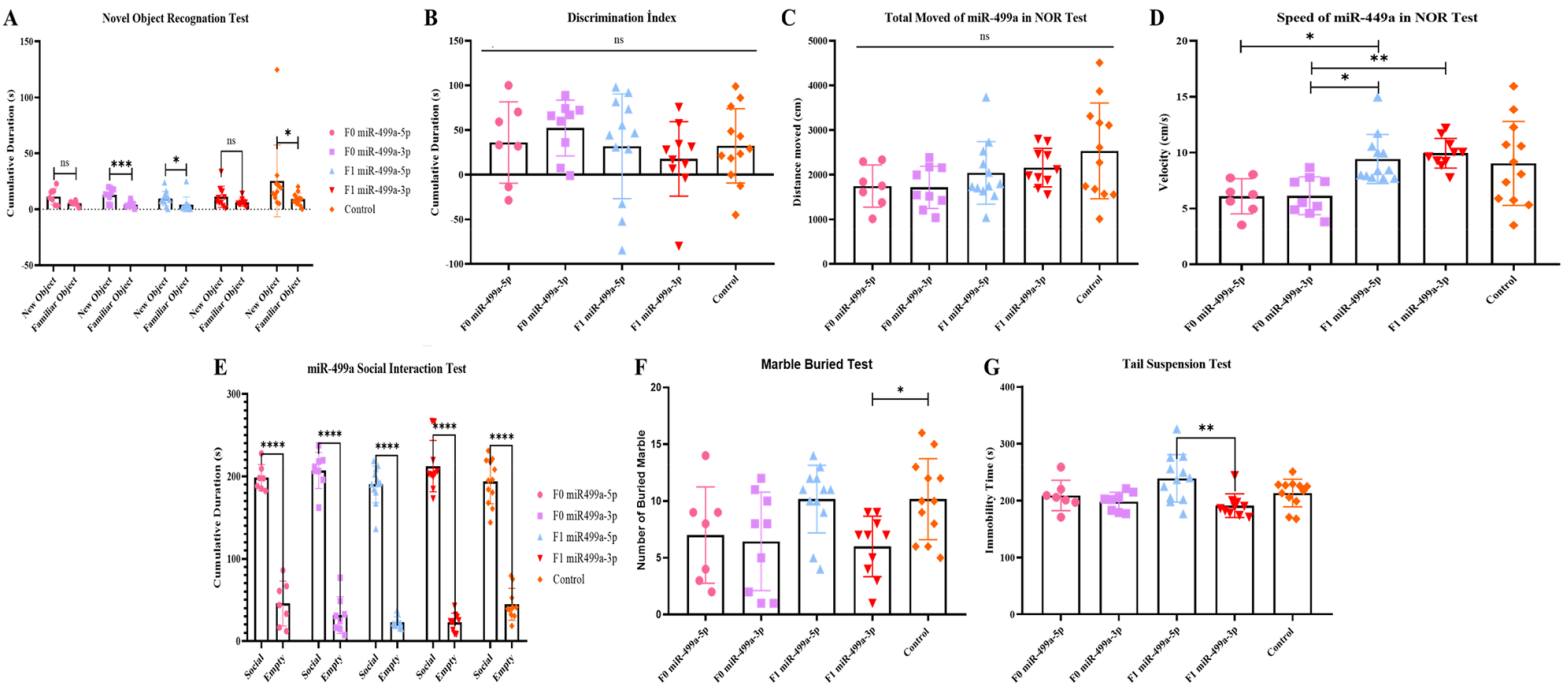
Behavior tests result of F0 generation mice group born after miR-499a microinjection (5p/3p) into fertilized mouse eggs (**A**) Comparison of total exploration time between new object and familiar object, (**B**) Comparison of total exploration time in F0 and F1 generations in NOR (**C**) Comparison of total movement between F0 and F1 generations in NOR test, (**D**) Comparison of total speed in NOR test, (**E**) Comparison of total social time, (**F**) Comparison of total buried number in F0 and F1 generations in Marble Test and (**G**) Comparison of immobility time in F0 and F1 generations in Tail Suspension Test. (All data were expressed with mean and ± SD; (N) = 7 for F0 mir499a-5p group, (N) = 9 for F0 miR499a-3p group, (N) = 12 for F1 mir499a-5p group, (N) = 10 for F1 miR499a-3p group and (N) = 15 for control).

Together all results of the behavioral tasks are shown in Figs. 6 and 7, with a number of variations observed mainly with certain less active groups of mice. Thus, the loss of interest between familiar objects and novel objects seems to indicate a common deficiency of the “autistic group” of mice comparable to the positive control group of male mice treated with Valproic acid as previously shown^5^.

As shown in Fig. 6A interest in the novel object increased in founder mice microinjected with both strands of miR-19a-5p and -3p, while their F1 generation showed a comparable phenotype to controls. In contrast, mice born after being microinjected with each strand of miR-499a-5p and -3p, now compared to the control group showed reduced interest in both objects observed for the founder as well as the F1 generation (Fig. 7A). Mice born after microinjection of miR-499a-5p and -3p into zygotes appear to behave with an autism-like phenotype comparable to autistic mouse models as we have previously reported with males treated with Valproic acid^5^.

The discrimination index, which measures the percentage of time spent with the novel object, shows that F1 miR-19a-3p spent significantly less time than the control group. In addition, we see that F0 miR-19a-5p spent less time than the other groups, while the time spent with the novel object was longer than the other groups. Similarly, F0 miR-19a-5p was found to be significantly more mobile and faster than all other groups. Even though there was a decrease in velocity in the F1 miR-19a-5p and -3p groups, it was observed that there was a significant increase compared to the control group. In the mice group born after microinjection with miR-499a, there was no significant difference between the groups in terms of discrimination index and total movement, while the group with the highest discrimination index was observed in F0 miR-499a-3p. This confirms the time spent with the novel object. In terms of speed, F1 miR-19a-5p and miR-19a-3p groups were observed to move faster than F0 miR-499a-3p and all other groups.

Social interaction or marble-burying test are frequently observed in the autistic mouse model. However, there were no or only very slight differences in social interaction and marble burying tests between the groups of mice microinjected with miR-19a or miR-499a. Although mice injected with mir-499a-5p or -3p showed signs of socializing behavior compared to mice injected with mir-19a-5p or 3p, this behavior was similar to the control. Likewise, although mice injected with mir-19a-5p or -3p showed less social and autistic behavior, this behavior was no different from controls (Figs. 6E–F and 7E–F).

The tail suspension experiment showed that autistic model mice develop a sedentary posture after the first escape movements when placed in an unavoidably stressful situation. Except for mice microinjected with miR-19a, on the other hand, all groups were more mobile than control, while only F1 showed significant difference between miR-19a-3p and miR-19a5p. Less mobility is observed in F0 miR-19a-3p within group. In mice microinjected with miR-499a-3p, F1 shows less activity than miR-499a-5p (see in Fig. 7). F1 miR-499a-3p is the least mobile of all groups. There is no significant difference between the other groups. In contrast to the novel object recognition test, the F1 miR-499-3p tail suspension test displayed more autistic behavior.

In conclusion, functional assays validate miRNA-mediated alteration of behavioral phenotypes.

### Decreased miRNA level impairs next generation: behavioral analysis reveals modified F1 generation animals

The expression levels of six miRNAs (-5p and -3p) in free fraction of the sperm of males born after microinjection of a specific miRNA are shown in Figure S3. The results of sperm miRNAs analysis indicate that microinjection of specific miRNAs (miR-499a-5p and -3p) dramatically reduced the expression levels of miR-19a-5p and miR-499a-3p (see in Figure S3B) and these were also found to be downregulated in our previous studies mouse models. Thus, increasing levels of these miRNAs by zygote microinjection leads to reduced levels of miRNAs in mouse sperm. Then, the F1 generation born to founding fathers showing downregulation of six miRNAs in sperm leads to offspring exhibiting variable behavioral manifestations, see Fig. 6.

### DNA-bound miRNA varies with changes in its own levels

**Figure 8:**
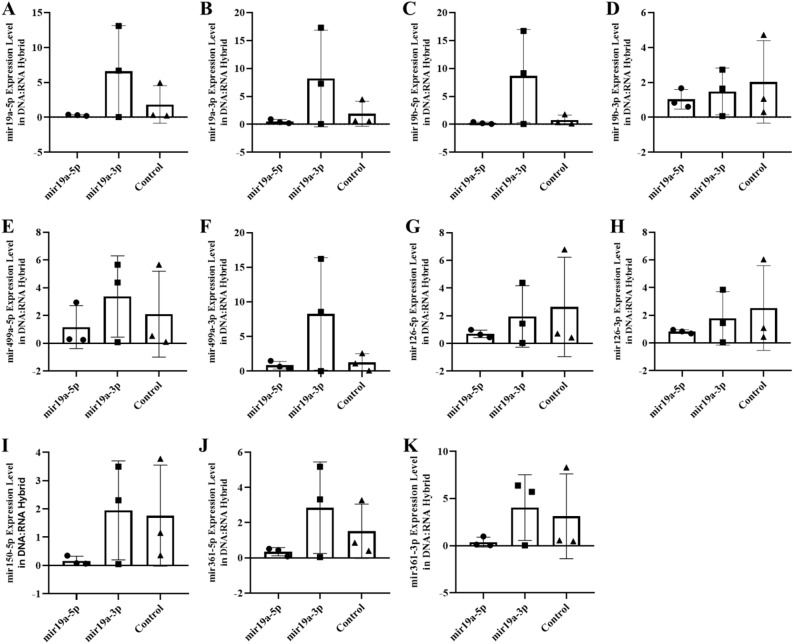
The expression of miRNAs (RNA fraction of DNA:RNA hybrid) in the blood of F0 generation mice born after microinjection of miR-19a (5p/3p) into fertilized mouse eggs. (**A**)* miR-19a-5p, *(**B**)* miR-19a-3p, *(**C**)* miR-19b-5p, *(**D**)* miR-19b-p , *(**E**)* miR-499a-5p, *(**F**)* miR-499a-3p, *(**G**)* miR-126a-5p, *(**H**)* miR-126a-3p, *(**I**)* miR-150a-5p , *(**J**)* miR-361a-5p and *(**K**)* miR-361a-3p* (All data were expressed with mean and ± SD; (N) = 5 for F0 mir19a-5p group, (N) = 5 for F0 miR19a-3p group and (N) = 5 for control; The x-axis illustrates the groups formed by microinjection with miR-19a-5p or -3p, alongside the control group. The y-axis represents the expressions of the miRNAs utilized in the study).

**Figure 9:**
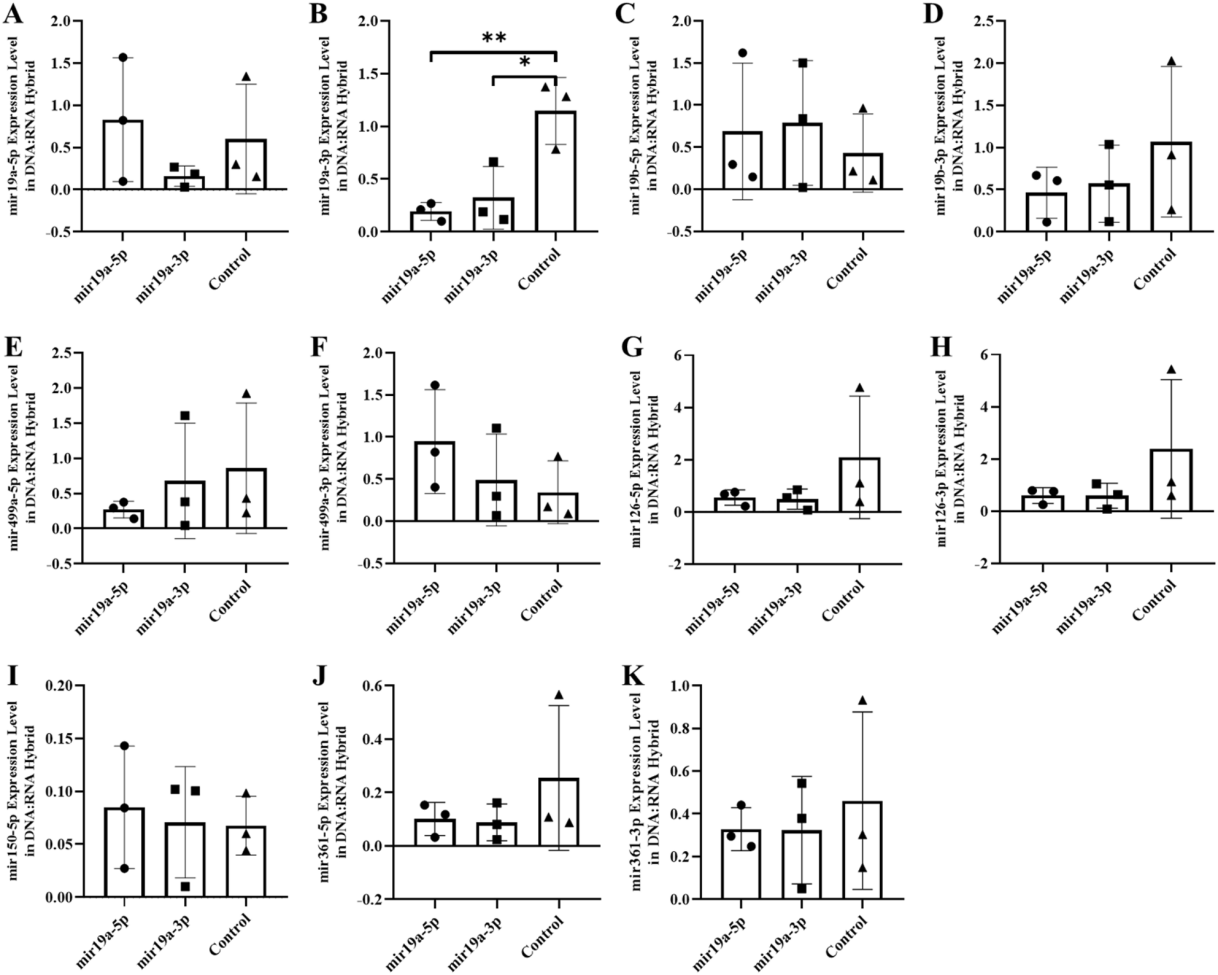
The expression of miRNAs (RNA fraction of DNA:RNA hybrid) in the hippocampus of F0 generation mice born after microinjection miR-19a(5p/3p) into fertilized mouse eggs. (**A**)* miR-19a-5p, *(**B**)* miR-19a-3p, *(**C**)* miR-19b-5p, *(**D**)* miR-19b-p, *(**E**)* miR-499a-5p, *(**F**)* miR-499a-3p, *(**G**)* miR-126a-5p, *(**H**)* miR-126a-3p, *(**I**)* miR-150a-5p, *(**J**)* miR-361a-5p and *(**K**)* miR-361a-3p* (All data were expressed with mean and ± SD; (N) = 5 for F0 mir19a-5p group, (N) = 5 for F0 miR19a-3p group and (N) = 5 for control; The x-axis illustrates the groups formed by microinjection with miR-19a-5p or -3p, alongside the control group. The y-axis represents the expressions of the miRNAs utilized in the study).

**Figure 10:**
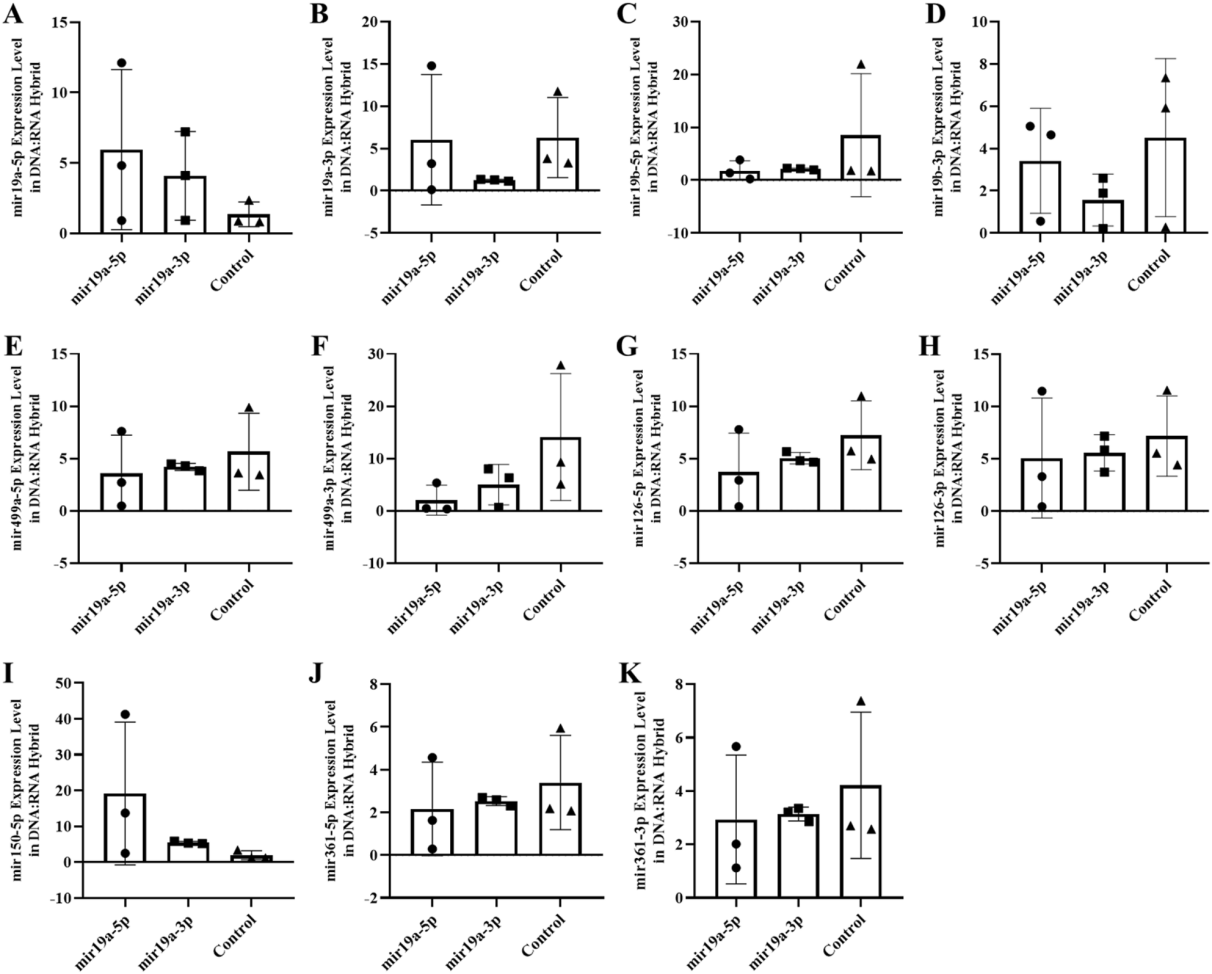
The expression of miRNAs ( RNA fraction of DNA:RNA hybrid) in sperm of F0 generation mice born after microinjection of miR-19a (5p/3p) into fertilized mouse eggs. (**A**)* miR-19a-5p, *(**B**)* miR-19a-3p, *(**C**)* miR-19b-5p, *(**D**)* miR-19b-p, *(**E**)* miR-499a-5p, *(**F**)* miR-499a-3p, *(**G**)* miR-126a-5p, *(**H**)* miR-126a-3p, *(**I**)* miR-150a-5p, *(**J**)* miR-361a-5p and *(**K**)* miR-361a-3p* (All data were expressed with mean and ± SD; (N) = 5 for F0 mir19a-5p group, (N) = 5 for F0 miR19a-3p group and (N) = 5 for control; The x-axis illustrates the groups formed by microinjection with miR-19a-5p or -3p, alongside the control group. The y-axis represents the expressions of the miRNAs utilized in the study)*.*

**Figure 11:**
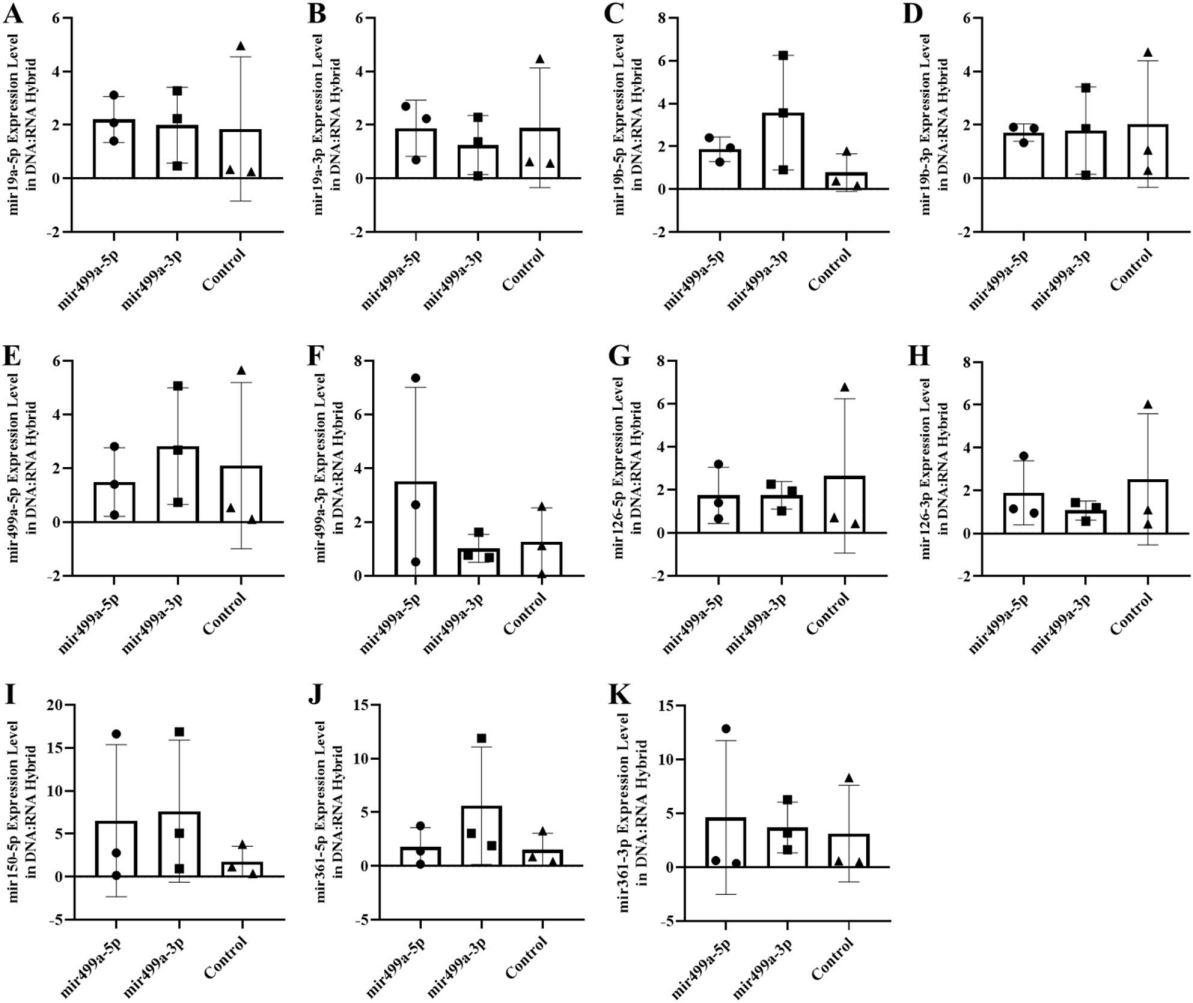
The expression of miRNAs (RNA fraction of DNA:RNA hybrid) in the blood of F0 generation mice born after microinjection of miR-499a(5p/3p) into fertilized mouse eggs. (**A**)* miR-19a-5p, *(**B**)* miR-19a-3p, *(**C**)* miR-19b-5p, *(**D**)* miR-19b-p, *(**E**)* miR-499a-5p, *(**F**)*. miR-499a-3p, *(**G**)* miR-126a-5p, *(**H**)* miR-126a-3p, *(**I**)* miR-150a-5p, *(**J**)* miR-361a-5p and *(**K**)* miR-361a-3p* (All data were expressed with mean and ± SD; (N) = 5 for F0 mir499a-5p group, (N) = 5 for F0 miR499a-3p group and (N) = 5 for control; The x-axis illustrates the groups formed by microinjection with miR-499a-5p or -3p, alongside the control group. The y-axis represents the expressions of the miRNAs utilized in the study).

**Figure 12:**
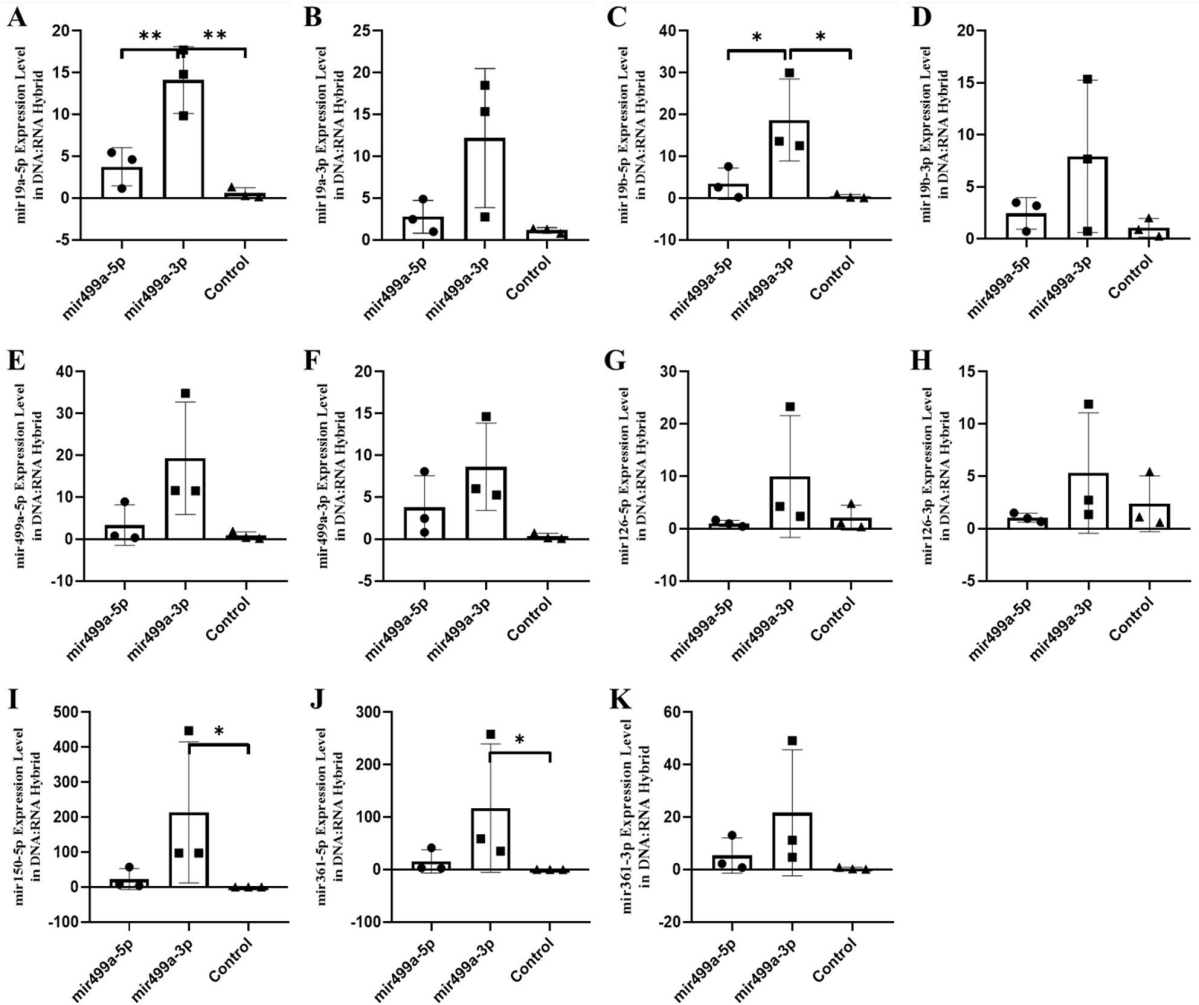
The expression of miRNAs (RNA fraction of DNA:RNA hybrid) in the hippocampus of F0 generation mice born after microinjection of miR-499a (5p/3p) into fertilized mouse eggs. (**A**)* miR-19a-5p, *(**B**)* miR-19a-3p, *(**C**)* miR-19b-5p, d. miR-19b-p, e. miR-499a-5p, f. miR-499a-3p, g. miR-126a-5p, h. miR-126a-3p, ı. miR-150a-5p, j. miR-361a-5p and k. miR-361a-3p* (All data were expressed with mean and ± SD; (N) = 5 for F0 mir19a-5p group, (N) = 5 for F0 miR19a-3p group and (N) = 5 for control; The x-axis illustrates the groups formed by microinjection with miR-499a-5p or -3p, alongside the control group. The y-axis represents the expressions of the miRNAs utilized in the study).

**Figure 13:**
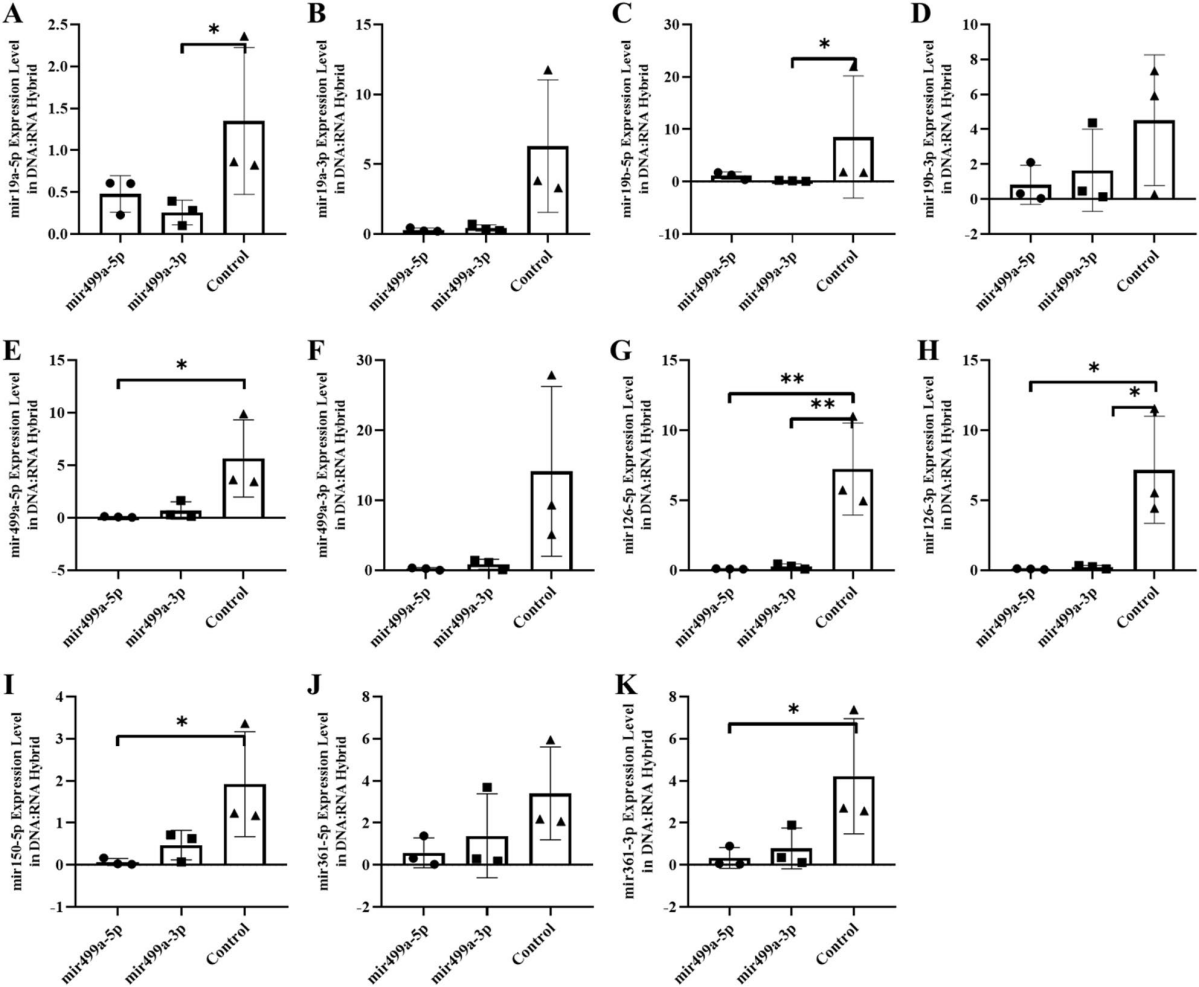
The expression of miRNAs (RNA fraction of DNA:RNA hybrid) in the sperm of F0 generation mice born after microinjection of miR-499a (5p/3p) into fertilized mouse eggs. (**A**)* miR-19a-5p, *(**B**)* miR-19a-3p, *(**C**)* miR-19b-5p, *(**D**)* miR-19b-p, *(**E**)* miR-499a-5p, *(**F**)* miR-499a-3p, *(**G**)* miR-126a-5p, *(**H**)* miR-126a-3p, *(**I**)* miR-150a-5p, *(**J**)* miR-361a-5p and *(**K**)* miR-361a-3p* (All data were expressed with mean and ± SD; (N) = 5 for F0 mir19a-5p group, (N) = 5 for F0 miR19a-3p group and (N) = 5 for control The x-axis illustrates the groups formed by microinjection with miR-499a-5p or -3p, alongside the control group. The y-axis represents the expressions of the miRNAs utilized in the study)*.*

In an abstract graphical summary, Figure S4 (blood), and Figure S5 (sperm) show the altered levels of the six miRNAs (-5p and -3p) in the free RNA fraction. We have previously reported that part of the nascent transcripts are retained on the genomic DNA in the hybrid structures^22^ and are not released by classical RNA extraction methods (TRIzol). Could the modification of the RNA level in the fraction attached to the DNA be affected? Thus, as the miRNA level is also changed and maintained at the different tissues level, we next investigated whether the decay of miRNAs in blood cells is also altered in the DNA-bound miRNAs fraction. RNAs retained on the hybrid structures (DNA/RNA) as R-Loop are extracted by extensive treatment with *Dnase* (see the Methods section). As expected, a strong reduction in miRNAs retained on the genome is observed in tissue samples from animals born after the microinjection of miRNA. However, the decay of six miRNAs was tissue-specific (blood, hippocampus and sperm). For example, the decay of miR-19a in blood samples was highly specific for miR-19a-5p with microinjection of miR-19a-5-p and at a significantly higher or unchanged level with miR-19a-3p microinjection (Fig. 8 blood). Results of mir19 are shown on the hippocampus and sperm (miR-19a) in Figs. 9 and 10 respectively. In contrast, decay in the hippocampus was specific for miR-499a-5p with miR-499a-5p microinjection and greater or unchanged with miR-499a-3p microinjection. Results of miR-499a are shown in Fig. 11 (blood), Fig. 12 (hippocampus), and Fig. 13 (sperm) respectively, see as well with graphical abstract Figure S4, S5 and S6).

**Figure 14:**
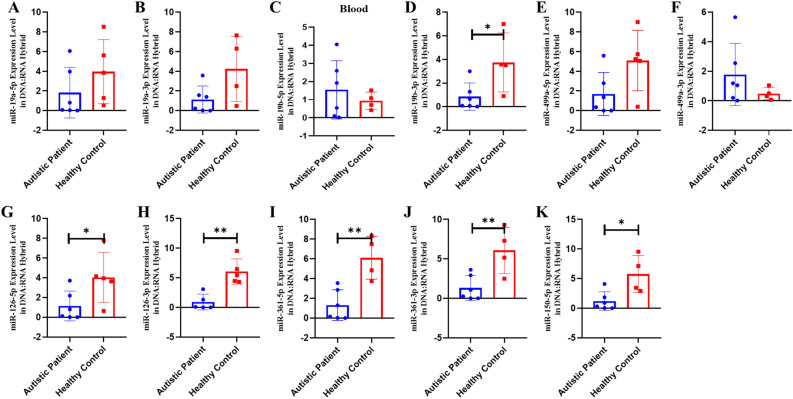
The expression of miRNAs of RNA fraction of DNA:RNA hybrid in the blood of human autistic patient. (**A**)* miR-19a-5p, *(**B**)* miR-19a-3p, *(**C**)* miR-19b-5p, *(**D**)* miR-19b-p, *(**E**)* miR-499a-5p, *(**F**)* miR-499a-3p, *(**G**)* miR-126a-5p, *(**H**)* miR-126a-3p, *(**I**)* miR-150a-5p, *(**J**)* miR-361a-5p and *(**K**)* miR-361a-3p* (All data were expressed with mean and ± SD; (N) = 6 for Autistic Patient group, (N) = 6 for Healthy Control group)*.*

To confirm and extend these results to human samples, we performed the same DNA-bound RNA extraction protocol from the blood cells of six patients or six controls. All six miRNAs (miR-19a-3p, miR-361-5p, miR-3613-3p, miR-150-5p, miR-126-3p and miR-499a-5p) including their -5p and 3p strand were also at lower levels in the DNA-bound RNA fraction of patient samples compared to controls in Fig. 14. Consistent with the animal results, most of the six miRNAs (-5p and -3p strands) showed a strong decrease and two (miR-499a-3p and miR-19b-5p) resisted to this decrease. A change in the distribution of all members of the six miRNAs was observed. The overall analysis further demonstrated that the decay selectivity of the miRNAs strand (DNA-bound RNA) in blood represent the six most highly downregulated miRNAs in these patient samples. In contrast, in patient blood samples miR-499a-3p (the opposite strand) remains elevated relative to the control. This analysis reiterates the tissue specificity and effect of 5p versus 3p miRNA sequences in the DNA-bound miRNA fraction (Fig. 14, see as well with graphical abstract Figure S7).

Together, these results show that the decay of the six miRNAs in DNA/RNA hybrids is a regulated decay process, rather than simply the result of synchronous decay through the post-transcriptional pathway since miRNAs levels of derivatives of the same transcript can be decoupled. Moreover, these results show that the sequence of the miRNAs is necessary for this regulated decay.

## Discussion

Despite overwhelming evidence for the level of transcription of miRNAs in normal development, the mechanism by which changes in the level (quantity) of these regulatory transcripts could lead to a heritable increase of the risk of pathologies is not understood. In patients and mouse models of autism, we reported^5^ that six miRNAs (miR-19a-3p, miR-361-5p, miR-3613-3p, miR-150-5p, miR-126-3p and miR-499a-5p) are downregulated. The aim of this study was to induce deregulation of specific miRNAs in serum and to assess phenotypic and transcriptional changes in mice. Here, we show that quantitatively changing the miRNAs levels in zygote pronuclei impact developing tissues. Now, we provide a direct method to modify experimentally the initial levels of two miRNA and nascent RNAs in the zygote.

Microinjection of each miRNA into zygotes, altered miRNA expression levels not only in serum, but also in hippocampus, correlated with behavioral changes and sperm. We confirmed that levels of serum miRNAs expression were lower in mouse models with affected behavior than in healthy controls. Interestingly, the regulation of miRNA expression depended on the type of miRNAs within the tested group which also showed transcriptional alterations already visible at their Pri- and Pre- miRNA levels. Each miRNA among the test groups when microinjected into zygote pronuclei induces a distinct phenotype and miRNA profile. The study of both strand (-5p and -3p) of two miRNAs in parallel makes it possible here to identify induced effects specific to each miRNA, for example miR-499a would induce phenotypic changes characteristic of the resilience of autism in mice while miR-19a would induce the opposite effects such as hyperactivity. Additionally, these results indicate that microinjection into the zygote of miRNAs could be regulators of their own transcriptions. At the same time, it is still being determined if this is a property of embryos or if any normal cells in the animal will react in the same way.

All results on miRNAs expression levels are summarized in Supplementary Figures S4, S5, and S6 (mouse) as a graphical abstract.

At this stage, known mechanisms do not easily explain how microinjection of miRNA into the zygote, which acts transiently in the embryo but modifies its transcription levels, triggers a cascade that will last a lifetime. As a result, the original pool of miRNA predicted to target the transcription networks of the developing embryo and the resulting transcription profile guide the reprogramming of RNA profiles in different tissues in a specific manner. Change in the miRNAs pool affect several organ development pathways^6–8^. Both genetic and environmental alterations have been shown to cause abnormal expression of miRNAs (see for review^23^). This downregulation after microinjection of a large quantity of miRNAs into the pronuclei of zygotes, is reminiscent of events reported in plants which depend on a threshold of small RNAs making it possible to establish and maintain specific communications between alleles^24^.

In accordance with our results, miRNA levels vary with environmental changes^25^. In particular, many studies show that the expression of miR-19 which belongs to miR-17-92a clusters, is highly affected by environmental factors^26,27^. In fact, miR-19 has been reported in at least three separate reports to be impaired in autistic patients^28^. Here, pronuclei of zygote artificially overloaded with miRNAs downregulated their transcription, which may alter the amounts of other miRNAs. We showed here that lower expression levels of each of the two miRNAs (miR-19a or miR-499a) were associated with behavioral changes. Indeed, in our data set, we found that changes in serum miRNA levels were associated with the recurrence of behavior changes.

Alterations in the level of miRNAs miR-19a or miR-499a participate in the variation of the phenotype. MiRNAs are deregulated in adult tissues when zygote pronuclei have been exposed to high level of miRNAs. Our current data prove that specific and distinct behavior changes accompanied the deregulation of two of miRNAs separately in the serum. These results suggest that miRNA levels are here regulated at an early stage of development.

Post-transcriptional regulatory mechanisms mediated by miRNAs at the levels of individual target transcripts are precisely detailed^10^. We have observed a relationship between the level of specific miRNAs (19a-, 499a-) and phenotypic changes, but the mechanisms by which the level of these two miRNAs are downregulated remains unknown. Several reports have shown that miRNAs are usually transcribed by RNA polymerase-II, but specific regulatory signals for expression are not known.

### DNA-bound miRNAs vary with alteration of miRNAs level

It is fundamental to learn how transgenerational transcript levels of RNA^29^ above all of miRNAs are regulated because a high number of coding and non-coding RNA are targeted by them. Elevated/decreased levels of a given miRNAs could be factored into overall changes in RNA-RNA, RNA-DNA and DNA/DNA interactions in the nucleus, but how they are stored and propagate for transfer to daughter cells, or the next generation is still uncertain. The organization of nuclear compartments also dependents on non-coding RNAs (ncRNAs)^30–33^.

Our data suggest that the regulatory mechanisms of the tested miRNAs differ from the cytoplasmic or TDMD post-transcription previously described for miRNAs pathways. First, decay is inherited as cellular memory from an altered stage of the one cell embryo. Second, the decrease is conserved in the fraction of the last transcription wave in sperm. Together, these data suggest that at least two of these six-miRNAs levels are initially regulated by their own quantity.

### Excess of miRNA in zygotes affect their levels of DNA/RNA hybrids

In eukaryotic cells, an individual promoter is used. The amount of transcripts which are produced is controlled from the initiation of transcription to post-transcription. Although the transcriptional potency of promoters, enhancers and silencers is variable, it does not account for how eukaryotes might allow immediate and simultaneous transcriptional variation. Simultaneous and efficient control of a gene transcription group is necessary for immediate co-transcription or silencing of functionally related transcripts.

Why are specific single-strand RNAs proposed as immediate signals for transcriptional variation? It results from the microinjection of small synthetic RNAs oligonucleotides (21–22 ribo-nucleotides) into zygote pronuclei. The reduction in RNA levels attached to DNA suggests that RNA regulates transcription levels through its homologous sequence and its interaction with DNA. Here, we report the induced variation in interaction of transcripts with the gene body. It is unclear if there is a minimal sequence length (here only 21 nucleotides) responsible for the regulatory impact. The multiplication of examples with complementary studies will demonstrate the precision by which the RNAs produced are specific regulatory element for their own synthesis.

Work in mice has now strongly suggested that miRNAs could regulate their own synthesis and in this way the amount of RNA could influence transcription. Here we show that downregulation is artificially induced by an excess of miRNA. Moreover, we find that associations of miRNAs with DNA create pathway-specific RNA expression that regulates the amount of miRNA with physiological impact. Importantly, we also observed the downregulation of all six miRNAs as free miRNA molecules and those attached to their own loci not only in mouse models but also in results correlated with the levels of miRNAs attached to DNA as hybrids molecules in patients with autism. We suggest a decay model of *six* miRNA*s* that vary the quantity of miRNAs attached to their loci.

These results show that transcriptional regulation of miRNA levels, which allows specific and/or simultaneous variation of RNA paralogs, would adjust the dynamic regulation of target RNA families. Transcript levels of mir-19a and miR-499a were altered; whether the strength of the impact of these changes is similar in the context of all miRNAs or RNAs sequences will be a matter of future investigation. Having not examined processing intermediates (not easy in oocytes), we still do not understand how alterations introduced into a miRNA affect its synthesis. We did not directly measure the levels of miRNA decay at the embryonic stage, leaving ambiguity as to the molecular basis for the up or downregulation of miRNA variants in the embryo. Moreover, the decay of miR-19a occurs at a rate similar to that of miR-499a; our interpretation is that these variants are subject to the same mechanism that targets DNA at its own locus.

## Conclusions

In conclusion, regulating a group of miRNAs in adult tissues depends on their quantity at an early stage of development. The miRNAs levels could be a potential prognostic marker and a therapeutic target in behavior diseases.

### Supplementary Information

Supplementary Information.

## Data Availability

All data generated or analysed during this study are included in this published article. Also, the datasets used and/or analysed during the current study available from the corresponding author on reasonable request.
