# Supplementary material for: Experimentally altering microRNA levels in embryos alters adult phenotypes
Source: Sci Rep. 2024 Aug 16;14:19014. doi: 10.1038/s41598-024-63692-7 (PMC11329699; doi:10.1038/s41598-024-63692-7)
Supplement: Supplementary file 1 — Supplementary Information. [file 41598_2024_63692_MOESM1_ESM.pptx]

## Slide 1
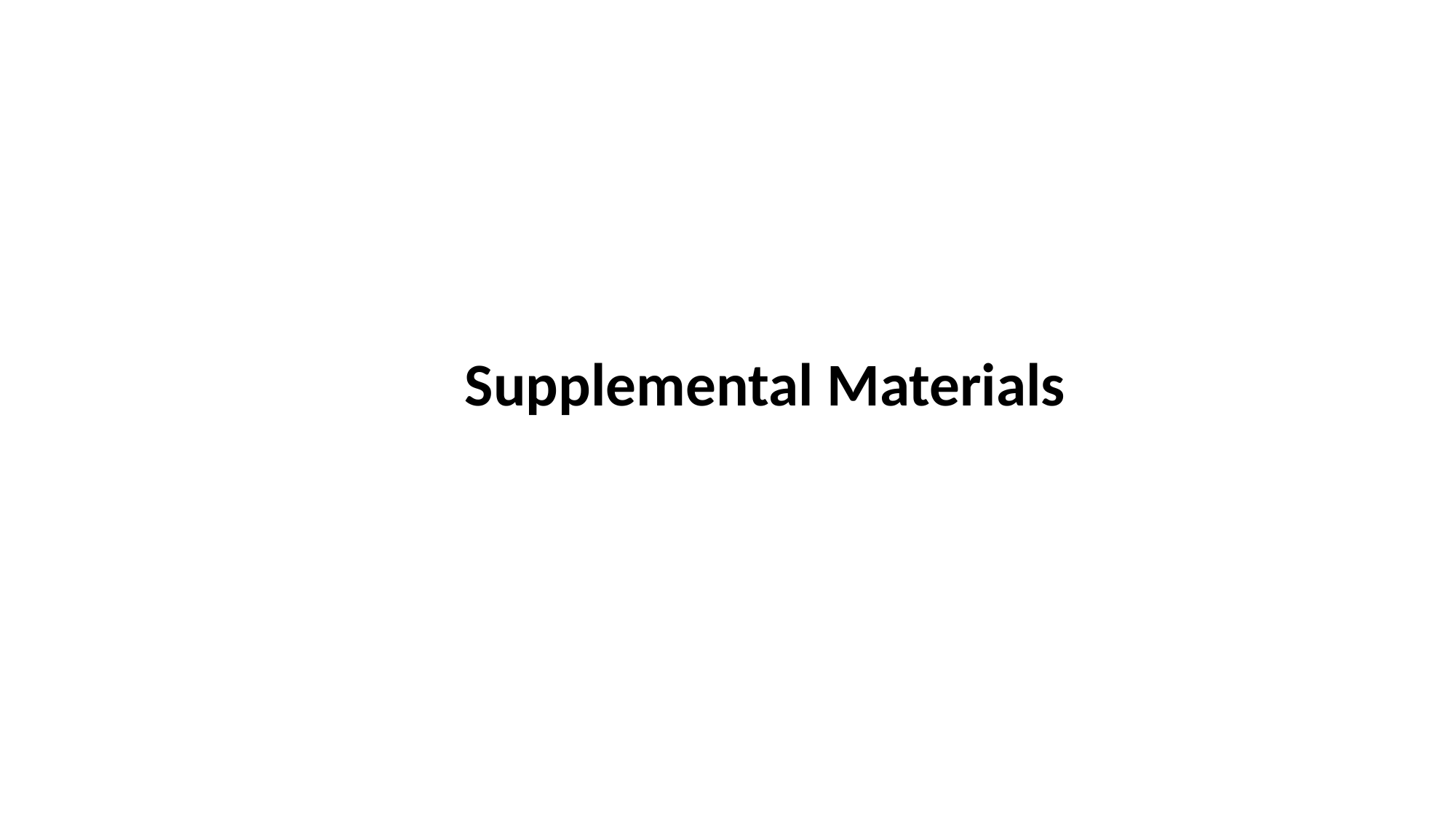

Supplemental Materials

## Slide 2
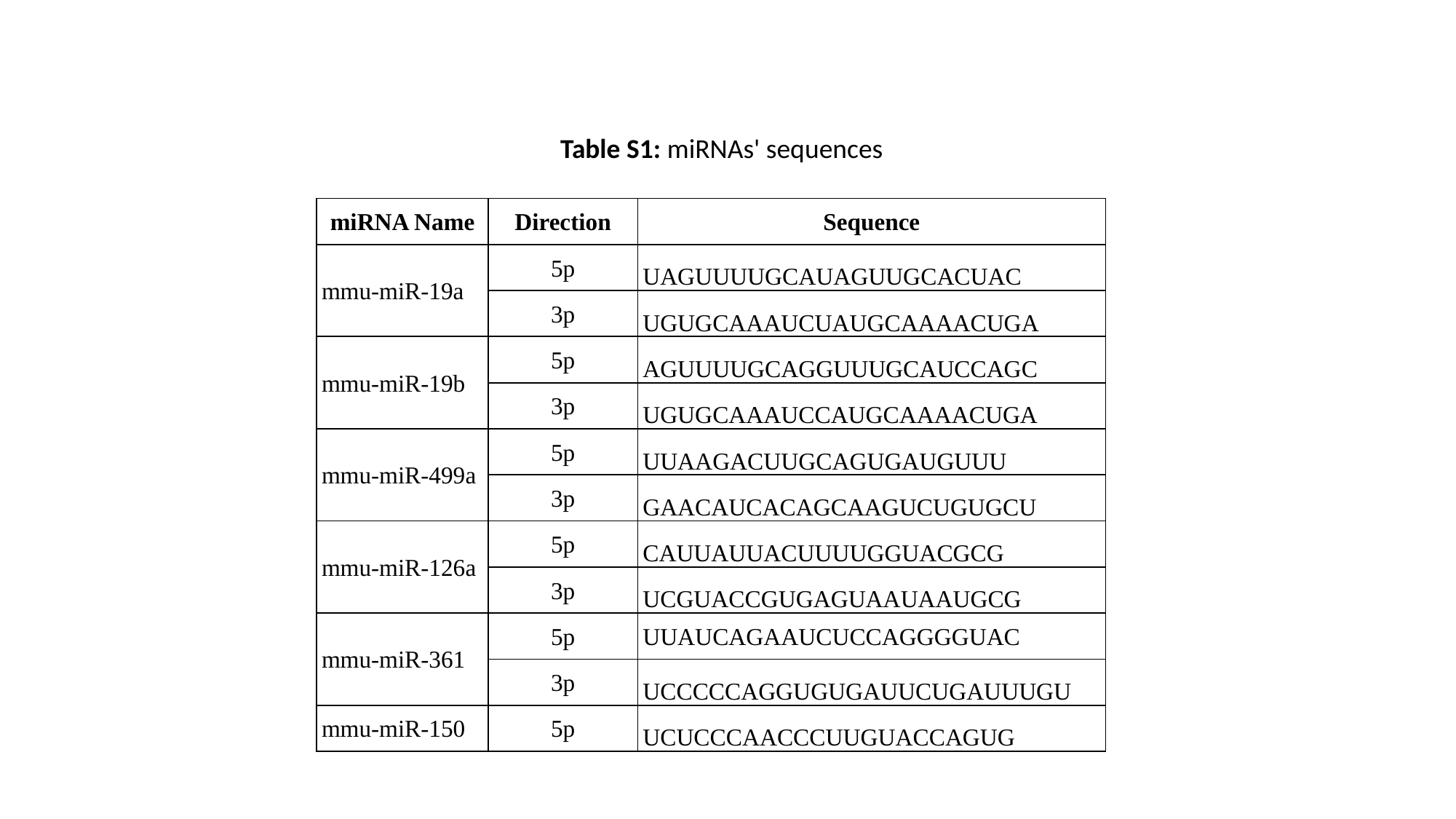

Table S1: miRNAs' sequences
| miRNA Name | Direction | Sequence |
| --- | --- | --- |
| mmu-miR-19a | 5p | UAGUUUUGCAUAGUUGCACUAC |
| | 3p | UGUGCAAAUCUAUGCAAAACUGA |
| mmu-miR-19b | 5p | AGUUUUGCAGGUUUGCAUCCAGC |
| | 3p | UGUGCAAAUCCAUGCAAAACUGA |
| mmu-miR-499a | 5p | UUAAGACUUGCAGUGAUGUUU |
| | 3p | GAACAUCACAGCAAGUCUGUGCU |
| mmu-miR-126a | 5p | CAUUAUUACUUUUGGUACGCG |
| | 3p | UCGUACCGUGAGUAAUAAUGCG |
| mmu-miR-361 | 5p | UUAUCAGAAUCUCCAGGGGUAC |
| | 3p | UCCCCCAGGUGUGAUUCUGAUUUGU |
| mmu-miR-150 | 5p | UCUCCCAACCCUUGUACCAGUG |

## Slide 3
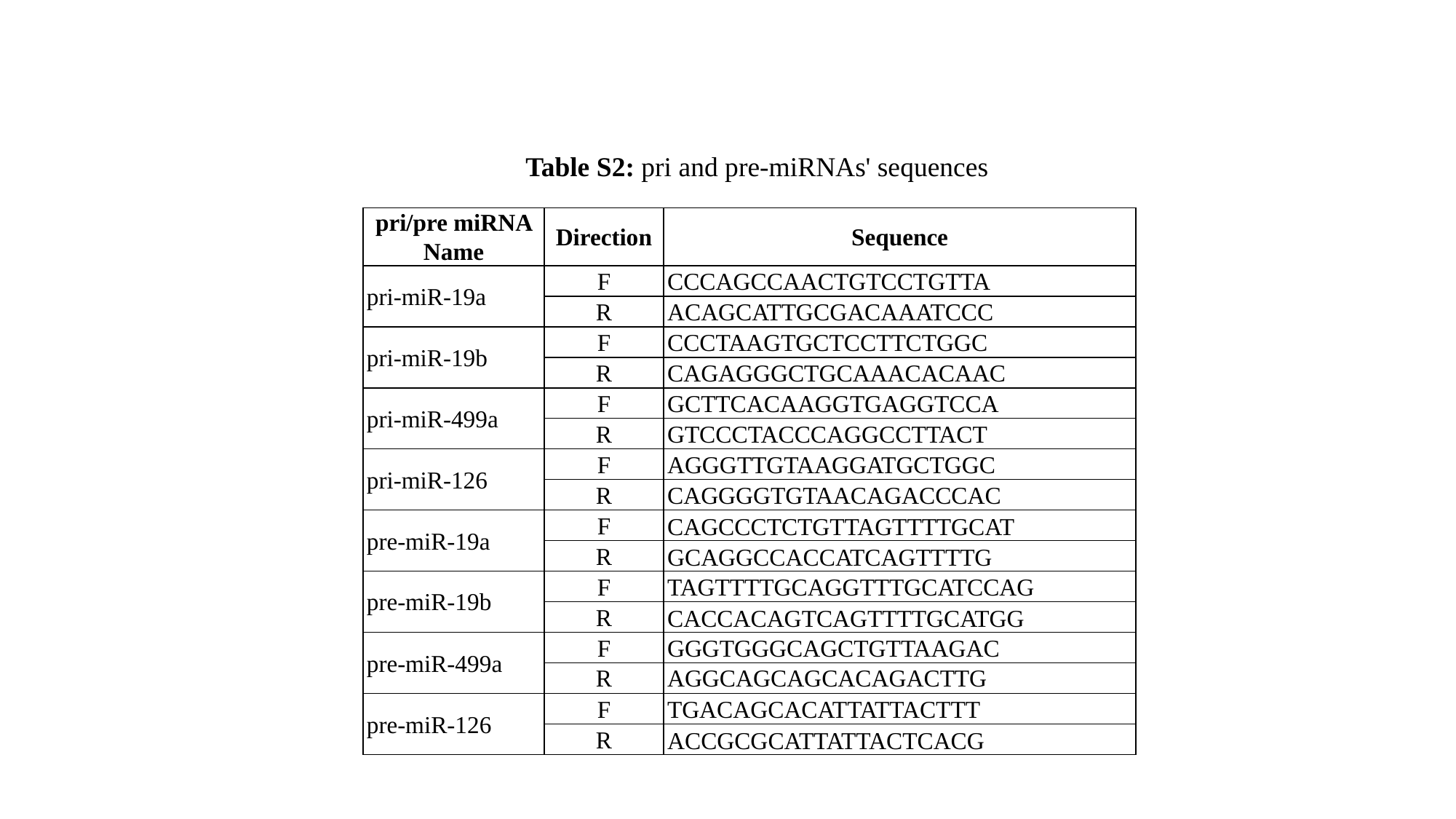

Table S2: pri and pre-miRNAs' sequences
| pri/pre miRNA Name | Direction | Sequence |
| --- | --- | --- |
| pri-miR-19a | F | CCCAGCCAACTGTCCTGTTA |
| | R | ACAGCATTGCGACAAATCCC |
| pri-miR-19b | F | CCCTAAGTGCTCCTTCTGGC |
| | R | CAGAGGGCTGCAAACACAAC |
| pri-miR-499a | F | GCTTCACAAGGTGAGGTCCA |
| | R | GTCCCTACCCAGGCCTTACT |
| pri-miR-126 | F | AGGGTTGTAAGGATGCTGGC |
| | R | CAGGGGTGTAACAGACCCAC |
| pre-miR-19a | F | CAGCCCTCTGTTAGTTTTGCAT |
| | R | GCAGGCCACCATCAGTTTTG |
| pre-miR-19b | F | TAGTTTTGCAGGTTTGCATCCAG |
| | R | CACCACAGTCAGTTTTGCATGG |
| pre-miR-499a | F | GGGTGGGCAGCTGTTAAGAC |
| | R | AGGCAGCAGCACAGACTTG |
| pre-miR-126 | F | TGACAGCACATTATTACTTT |
| | R | ACCGCGCATTATTACTCACG |

## Slide 4
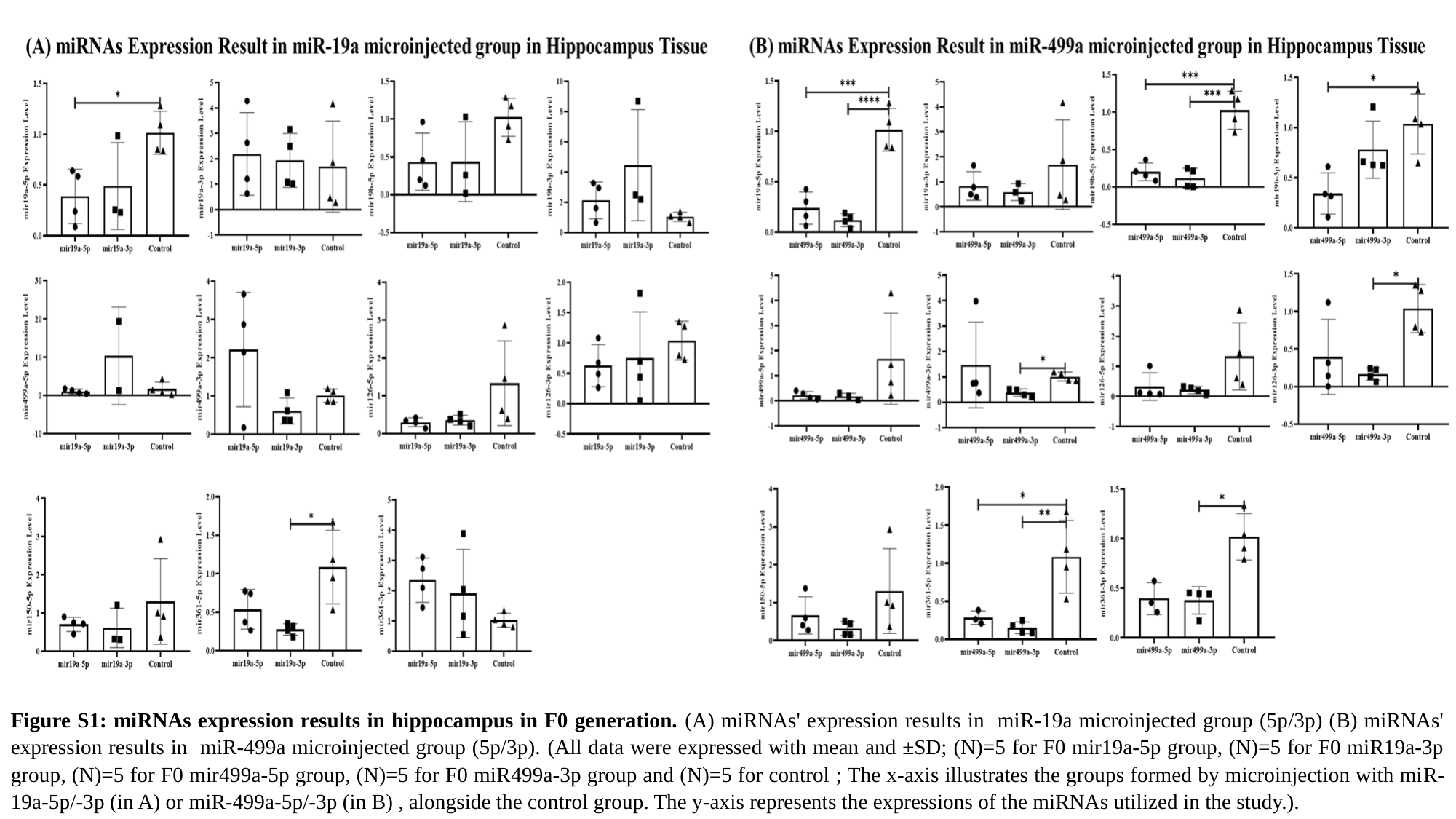

Figure S1: miRNAs expression results in hippocampus in F0 generation. (A) miRNAs' expression results in miR-19a microinjected group (5p/3p) (B) miRNAs' expression results in miR-499a microinjected group (5p/3p). (All data were expressed with mean and ±SD; (N)=5 for F0 mir19a-5p group, (N)=5 for F0 miR19a-3p group, (N)=5 for F0 mir499a-5p group, (N)=5 for F0 miR499a-3p group and (N)=5 for control ; The x-axis illustrates the groups formed by microinjection with miR-19a-5p/-3p (in A) or miR-499a-5p/-3p (in B) , alongside the control group. The y-axis represents the expressions of the miRNAs utilized in the study.).

## Slide 5
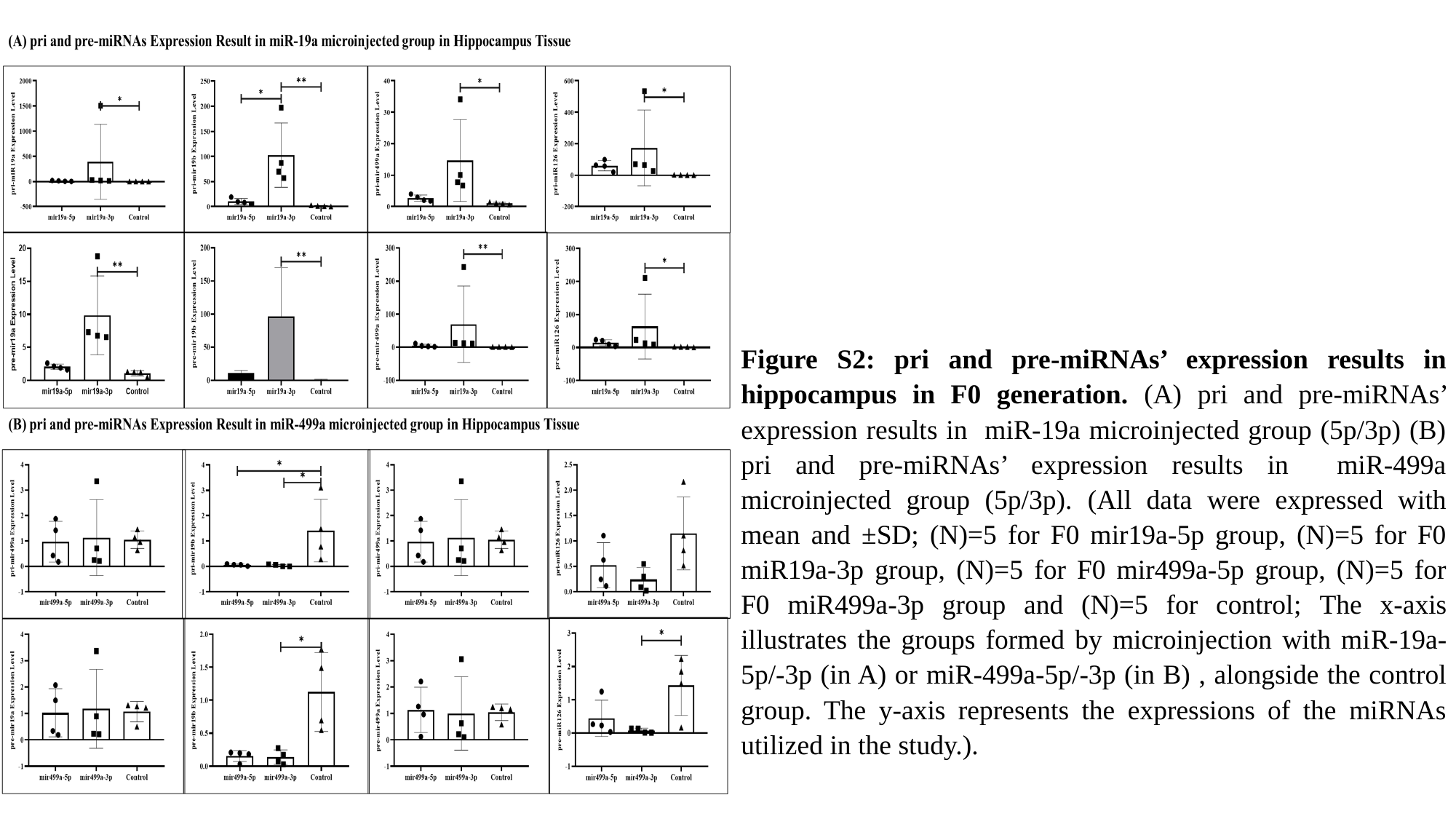

Figure S2: pri and pre-miRNAs’ expression results in hippocampus in F0 generation. (A) pri and pre-miRNAs’ expression results in miR-19a microinjected group (5p/3p) (B) pri and pre-miRNAs’ expression results in miR-499a microinjected group (5p/3p). (All data were expressed with mean and ±SD; (N)=5 for F0 mir19a-5p group, (N)=5 for F0 miR19a-3p group, (N)=5 for F0 mir499a-5p group, (N)=5 for F0 miR499a-3p group and (N)=5 for control; The x-axis illustrates the groups formed by microinjection with miR-19a-5p/-3p (in A) or miR-499a-5p/-3p (in B) , alongside the control group. The y-axis represents the expressions of the miRNAs utilized in the study.).

## Slide 6
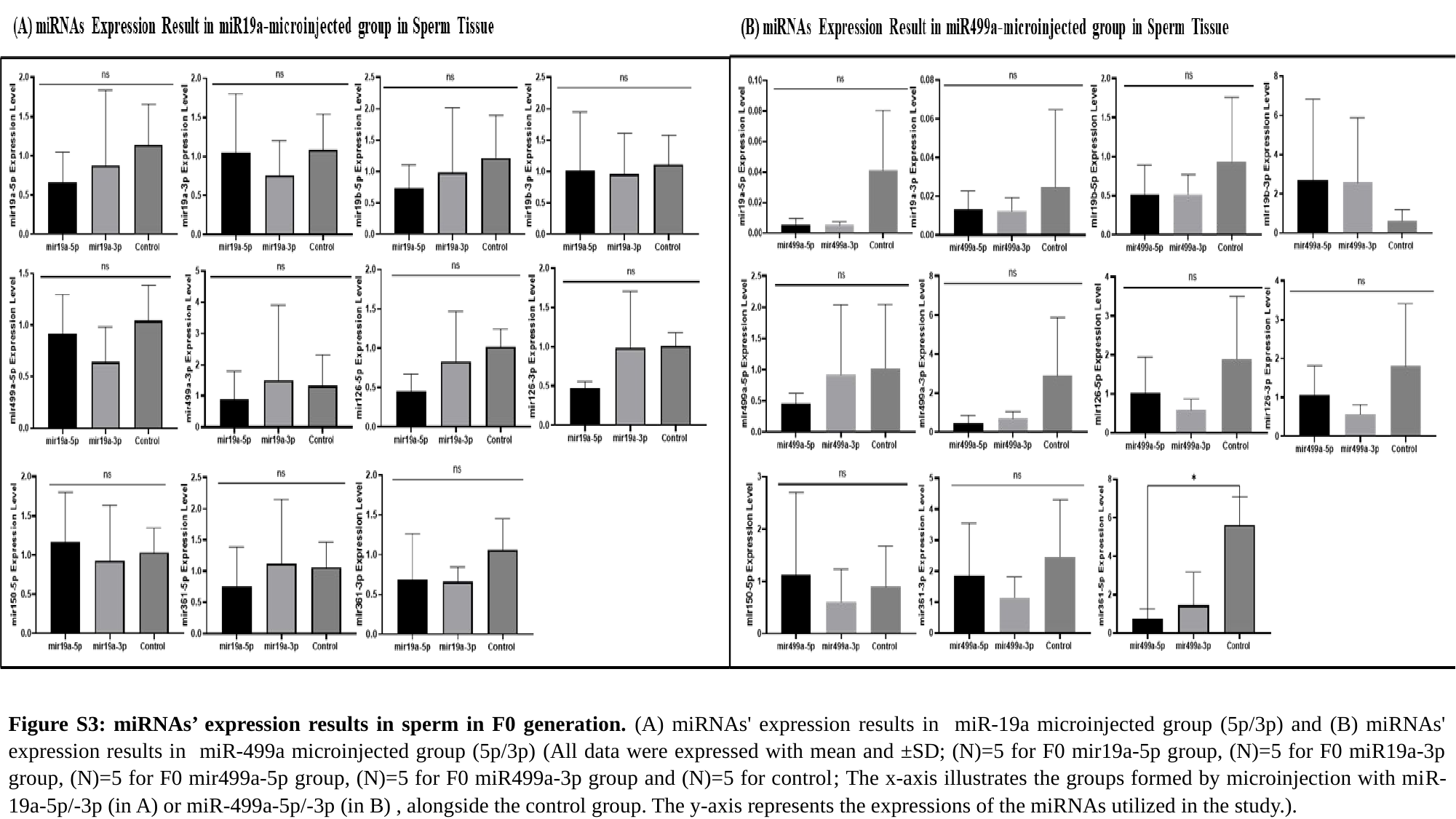

Figure S3: miRNAs’ expression results in sperm in F0 generation. (A) miRNAs' expression results in miR-19a microinjected group (5p/3p) and (B) miRNAs' expression results in miR-499a microinjected group (5p/3p) (All data were expressed with mean and ±SD; (N)=5 for F0 mir19a-5p group, (N)=5 for F0 miR19a-3p group, (N)=5 for F0 mir499a-5p group, (N)=5 for F0 miR499a-3p group and (N)=5 for control; The x-axis illustrates the groups formed by microinjection with miR-19a-5p/-3p (in A) or miR-499a-5p/-3p (in B) , alongside the control group. The y-axis represents the expressions of the miRNAs utilized in the study.).

## Slide 7
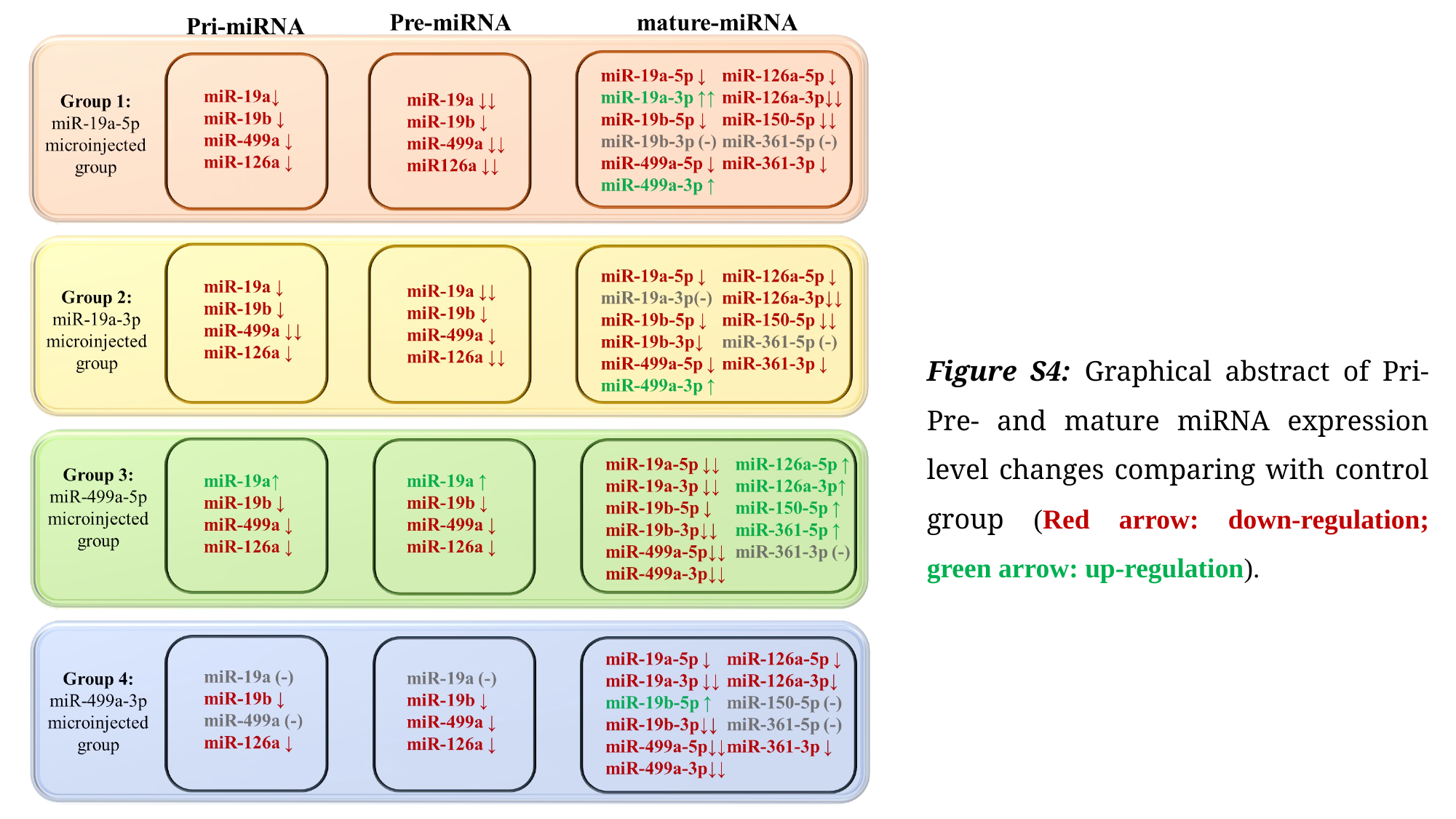

Figure S4: Graphical abstract of Pri-Pre- and mature miRNA expression level changes comparing with control group (Red arrow: down-regulation; green arrow: up-regulation).

## Slide 8
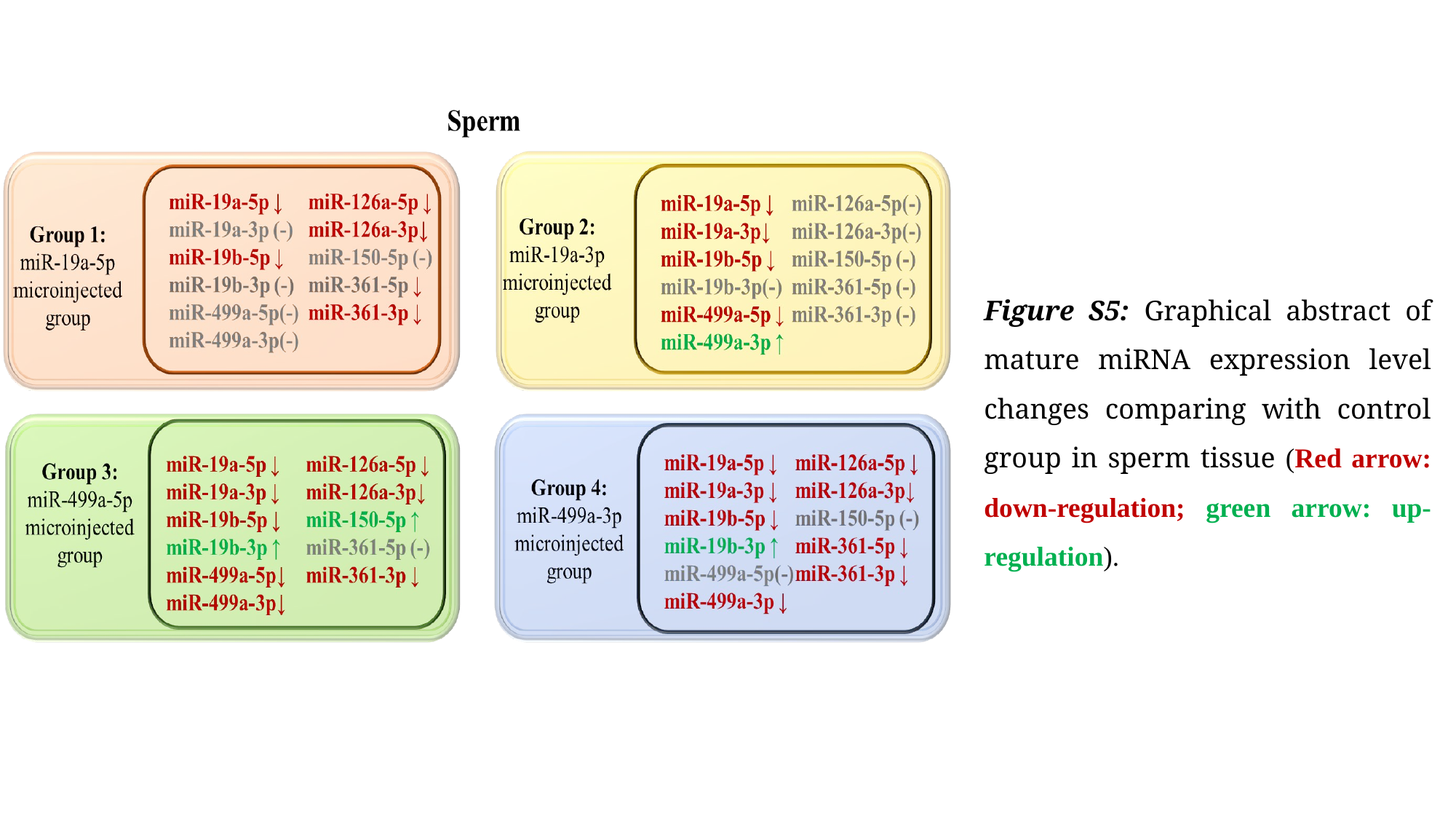

Figure S5: Graphical abstract of mature miRNA expression level changes comparing with control group in sperm tissue (Red arrow: down-regulation; green arrow: up-regulation).

## Slide 9
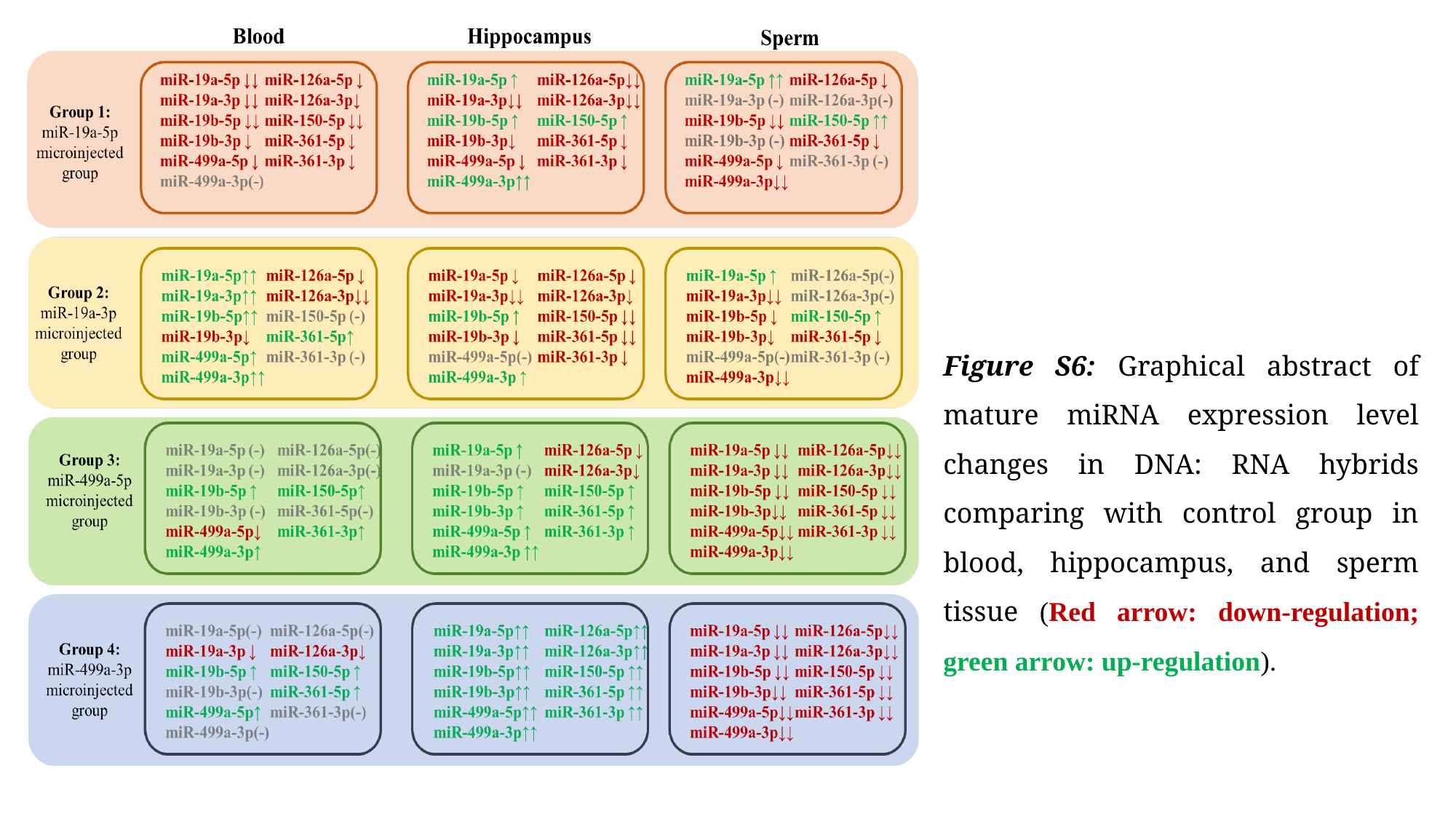

Figure S6: Graphical abstract of mature miRNA expression level changes in DNA: RNA hybrids comparing with control group in blood, hippocampus, and sperm tissue (Red arrow: down-regulation; green arrow: up-regulation).

## Slide 10
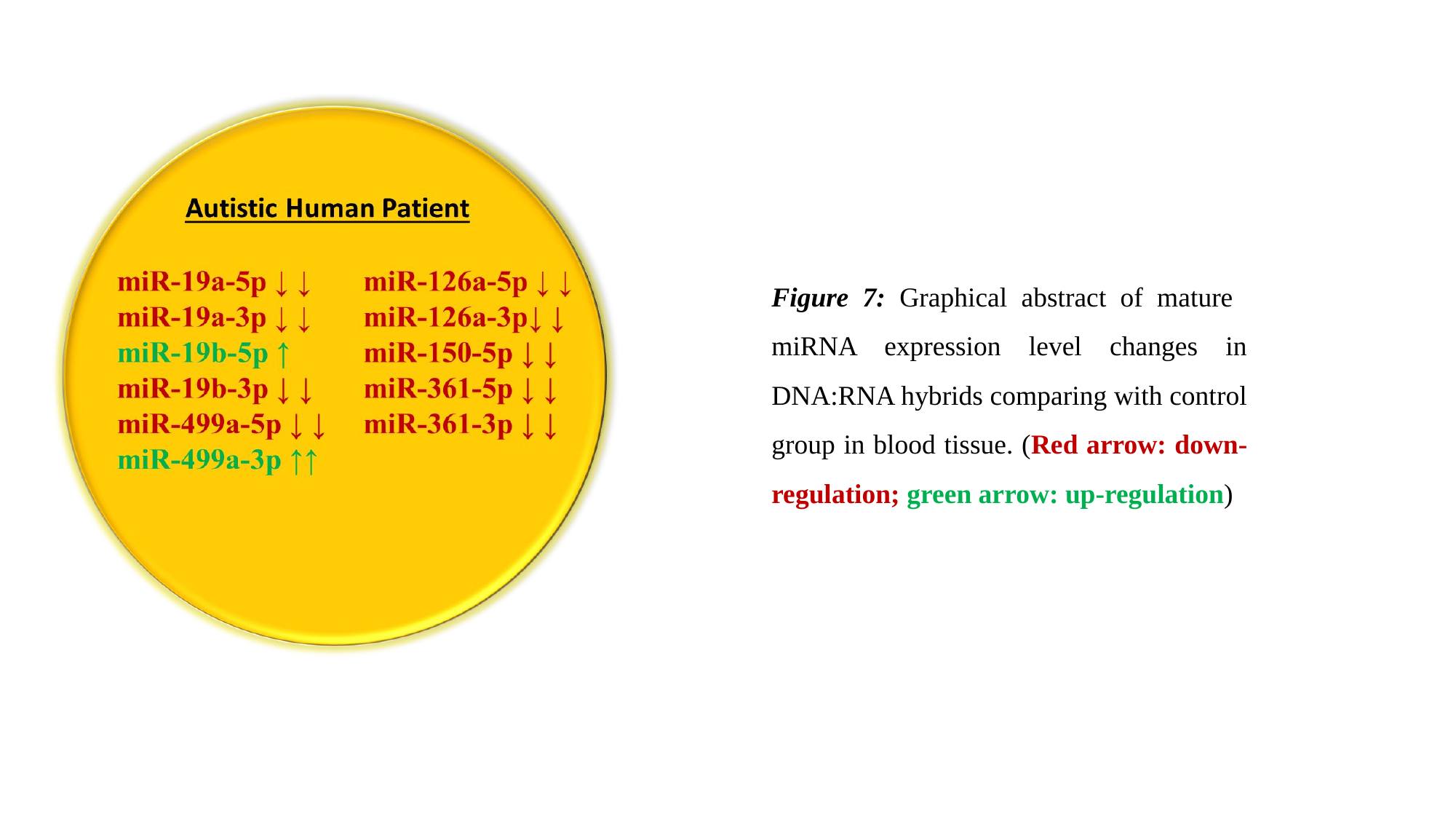

Figure 7: Graphical abstract of mature miRNA expression level changes in DNA:RNA hybrids comparing with control group in blood tissue. (Red arrow: down-regulation; green arrow: up-regulation)
